# Independent Control of Elastomer Properties through Stereocontrolled Synthesis

**DOI:** 10.1002/anie.201606750

**Published:** 2016-10-06

**Authors:** Craig A. Bell, Jiayi Yu, Ian A. Barker, Vinh X. Truong, Zhen Cao, Andrey V. Dobrinyin, Matthew L. Becker, Andrew P. Dove

**Affiliations:** ^1^ Department of ChemistryThe University of WarwickCoventryCV4 7ALUK; ^2^ Department of Polymer ScienceThe University of AkronAkronOH44325USA

**Keywords:** click chemistry, elastomers, organocatalysis, step-growth polymerization, stereochemistry

## Abstract

In most synthetic elastomers, changing the physical properties by monomer choice also results in a change to the crystallinity of the material, which manifests through alteration of its mechanical performance. Using organocatalyzed stereospecific additions of thiols to activated alkynes, high‐molar‐mass elastomers were isolated via step‐growth polymerization. The resulting controllable double‐bond stereochemistry defines the crystallinity and the concomitant mechanical properties as well as enabling the synthesis of materials that retain their excellent mechanical properties through changing monomer composition. Using this approach to elastomer synthesis, further end group modification and toughening through vulcanization strategies are also possible. The organocatalytic control of stereochemistry opens the realm to a new and easily scalable class of elastomers that will have unique chemical handles for functionalization and post synthetic processing.

Nature has evolved the ability to create large and complex molecules in which the precise control over the spatial arrangement of the atoms is critical to their performance. The three‐dimensional control over the arrangement of bonds is as important to the function and behavior of molecules as any other factor and is critical to the structure–function relationships that govern the role of a range of molecules. While the effect of stereochemistry on functionality is probably best known in the examples of small molecule drugs such as thalidomide (one enantiomer is effective against morning sickness, the other is teratogenic) or naproxen (one enantiomer is used to treat arthritis pain, the other causes liver poisoning with no analgesic effect), it is less well‐studied with respect to materials design.

Elastomeric materials are applied widely to demanding applications on account of their inherent reversible deformation behavior. Many synthetic elastomer materials are tri‐ or multi‐block copolymers that are based on the concept of an amorphous‐crystalline or hard–soft phase‐separated system in which organization of the hard and soft domains endows the strong but elastic properties upon the materials.^1^ While these materials have found widespread application, changes to the monomers or stoichiometry designed to elicit a change in physical properties also alter the chain packing and hence the mechanical properties of the materials. Interestingly, natural rubber and gutta percha are homopolymers of poly(*cis*‐isoprene) and poly(*trans*‐isoprene) respectively. While these materials differ by only the double bonds of the backbone, the superior elastomeric properties of natural rubber^2^ are attributed to the enhanced chain packing afforded by its stereochemical orientation.^3^ While the design principles to control crystallinity and the associated mechanical properties in these materials are clear, the inability to incorporate a wide range of functional groups in a controlled manner or rationally define the chain end functionality limits the applications of both these materials and their synthetic analogues.^4^ Combination of the control over degree of crystallinity and mechanical properties through backbone double‐bond stereochemistry, as exemplified by natural rubber/gutta percha, with the tunability of materials properties of synthetic materials via monomer selection and end group modification, presents a new method by which to undertake the rational design of elastomers and presents an entire new design space for functional materials.

A critical deficiency of synthetic methods in the ability to control double‐bond stereochemistry using monomers with diverse functional groups limits the development of novel materials that mimic the method of mechanical property control exemplified in natural rubber and gutta percha elastomers. The application of click chemistry, reactions that are so categorized because of their modular nature, single reaction trajectory, equimolarity, high yields, simple purification, chemoselectivity, and fast timescales,^5^ could potentially address the synthetic pitfalls and afford access to high‐molar‐mass polymers with defined stereochemistry. Indeed, we and others have recently demonstrated that the organobase‐catalyzed addition of thiols to activated alkynes could be stopped after a single addition and furthermore, by judicious choice of catalyst or solvent polarity, the stereochemistry of the resultant alkene could be controlled.^6^ Application of this method to step‐growth polymerization has allowed us to access high molar mass polymers with control over double bond stereochemistry, monomer composition, and chain‐end functionality to create elastomeric materials with independently tuneable mechanical and physical properties.

To access suitable materials, a dialkyne monomer (C_3A_) synthesized by simple Fischer esterification of propiolic acid and propane diol was combined with hexane dithiol (C_6S_) in chloroform (CHCl_3_) in the presence of 1 mol % of 1,8‐diazabicycloundecane (DBU) to promote *cis* double‐bond formation (Scheme 1). After only 1 h, precipitation of the polymer into diethyl ether revealed the formation of a material with high molar mass (Supporting Information, Figure S1 and S2; Tables S3 and S4) with a high *cis* content (80 %). Interestingly, changing the polarity of the reaction solvent by blending CHCl_3_ with *N*,*N*‐dimethylformaldehyde (DMF) and using 1 mol % of trimethylamine (NEt_3_) enabled the synthesis of materials with altered *cis*/*trans* ratios (Supporting Information, Figure S3). Further analysis of the materials by size‐exclusion chromatography using viscometry detection (Supporting Information, Figure S4) revealed *α* values of about 0.6, which is highly characteristic of a linear, unbranched, or non‐cross‐linked material in a good solvent.^7^

**Scheme 1:**
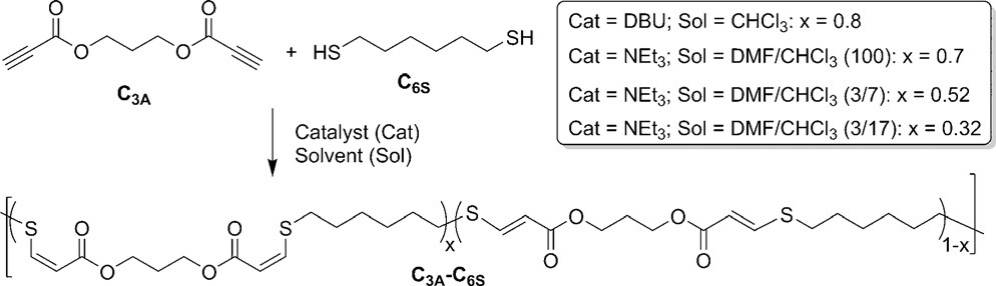
Synthesis of thiol–yne elastomer materials from dialkyne and dithiol precursors.

Analysis of the materials by wide‐angle X‐ray diffraction (WAXD) analysis revealed that while the 80 % *cis* material displayed sharp 2*θ* peaks at 21 and 23°, these peaks were significantly diminished in the 70 % *cis* polymers. Materials at lower % *cis* double bonds display broad peaks that are consistent with amorphous materials that exhibit no strong crystalline domains (Figure 1 A). Complimentary analysis by small‐angle X‐ray scattering (SAXS) confirms these observations with microcrystalline domains of ca. 18 and 22 nm (calculated from the *q* value at maximum intensity) being observed for the 80 and 70 % *cis* materials, respectively. Again, at lower %*cis* bond contents, no significant peaks were observed in the SAXS analysis (Figure 1 B). Differential scanning calorimetry (DSC) revealed, along with glass transitions of the materials at about −2 °C, that the 80 % *cis* material displays melting transitions in both the first and second scan data (Figure 1 C). A reduction in the % *cis* content to 70 % resulted in the observation of a melting peak in only the first scan which had a decreased peak area that corresponds to decreased crystallinity within the sample. Additional decreases in the % *cis* content further decreased the crystallinity of the sample. These data are confirmed by the diffraction data and indicate that the ordering of the polymer chains into microcrystalline domains is enhanced by high *cis* contents, in line with that of natural and synthetic polyisoprene and its analogues. Interestingly, the WAXD and SAXS spectra of the 80 % *cis* material after stretching revealed a significant change in the crystallinity of the materials consistent with strain induced crystallization (Supporting Information, Figure S5).

**Figure 1:**
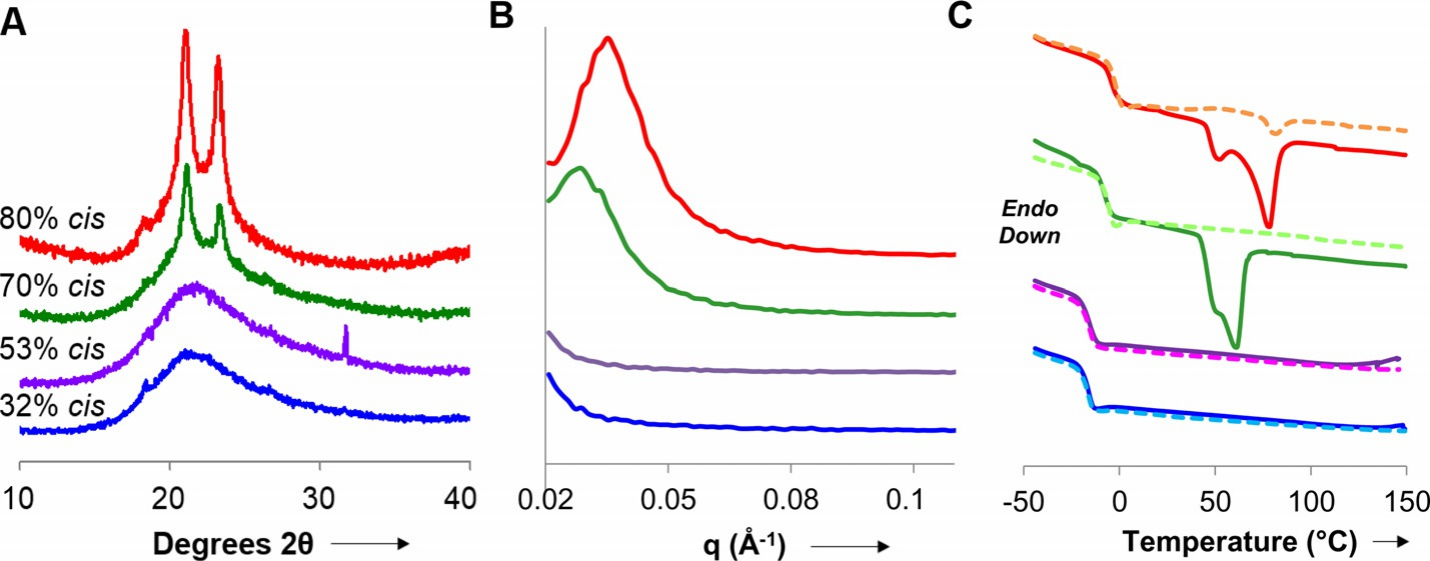
A) Wide‐angle X‐ray diffraction, B) small‐angle X‐ray scattering, and C) differential scanning calorimetry (solid=first scan; dashed=second scan) for C_3A_–C_6S_ materials.

The semicrystalline nature of the networks manifests itself in a significant improvement of the network mechanical properties with increasing fraction of the *cis* isomers (Figure 2 A). Investigation of the elastomer mechanical properties was undertaken to quantify deformation over a wide range of strain rates (2.34×10^−6^ to 9.38×10^−2^ s^−1^). The data sets collapse into a straight line which indicates that for the studied time interval the network mechanical properties are time (deformation rate) independent. To obtain the equilibrium Young's modulus at small deformations, the data set was fitted by the linear function *σ*_true_(*t*)/$\dot{\epsilon }$ =*E*_0_ *ϵ*(*t*)/$\dot{\epsilon }$ . From the resultant values of the Young's modulus (Table 1, Figure 2 B), the networks can be concluded to function as composite materials for which modulus depends on the network degree of crystallinity *φ*_c_. Using a mixture rule, the Young's modulus of the materials as a function of their degree of crystallinity is expressed as *E*_0_=(1−*φ*_c_)*E*_a_+*φ*_c_ *E*_c_, where *E*_a_ is Young's modulus of the amorphous phase and *E*_c_ is the Young's modulus of the crystalline phase. The modulus of the amorphous phase, *E*_a_, is estimated by averaging the modulus of samples with 32 % and 53 % *cis* isomer content, resulting in *E*_a_=2.94 MPa. In turn, the modulus of the crystalline phase, *E*_c_, from fitting the data is equal to 238.4 MPa. This value shows significant improvement over the mechanical properties of existing natural or synthetic elastomer analogues.

**Figure 2:**
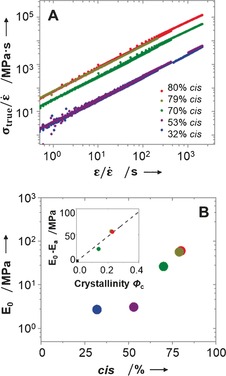
A) Dependence of the normalized network true stress *σ*_true_(*t*)/$\dot{\epsilon }$ as a function of time *ϵ*(*t*)/$\dot{\epsilon }$ for networks with different fraction of the *cis*‐isomers obtained from the constant strain rate experiments (2.34×10^−6^ to 9.38×10^−2^ s^−1^); B) Dependence of the network Young's modulus on the fraction of *cis* isomers. Inset: the linear dependence of *E*_0_−*E_a_* on the degree of crystallinity *φ*_c_.

**Table 1:** Mechanical properties of thiol–yne elastomers (C_3A_–C_6S_).

% *cis*	Crystallinity [%]	*E*_0_ [MPa]	*ϵ*_max_^[a]^ [%]	*ϵ*_break_ [%]	Tensile toughness^[b]^ [MJ *m* ^−3^]	UTS [MPa]
80	22.3	59.8±0.2	–	1495±66	289±45	54.3±6.5
79	22.9	56.5±6.4	–	1554±71	330±29	56.7±5.2
70	14.1	26.2±0.3	–	1874±94	292±58	43.5±6.5
53	0	3.1±0.1	346	2252±115	104±22	16.6±3.8
32	0	2.7±0.1	378	2970±137	35±3	2.8±0.4

[a] *ϵ*_max_ estimated from the value of the Young's modulus at small deformations. [b] Network toughness is calculated at a strain rate of 2.34×10^−2^ s^−1^.

Further analysis of these data using a value of the shear modulus, *G*=*E*_0_/3, of the network in the amorphous phase enables the estimation of the maximum elongation ratio. The molecular mass of strands supporting stress (*M*) is estimated from the network shear modulus as *G*≈*ρRT*/*M*→*M*≈*ρRT*/*G*, where *ρ* is density (assumed to be 1.0 g cm^−3^ for all materials in this study), *R* is the gas constant, and *T* is the absolute temperature. Furthermore, the contour length of the chain can be determined as *L*_0_=*l*_0_ *M*/*M*_0_, where *M*_0_ is monomer molecular mass (330.10 g mol^−1^) and *l*_0_ is the length of monomer in *cis*/*trans* configuration (Supporting Information, Figure S12, Table S2). The maximum extension ratio, *λ*_max_≈$\sqrt{l{}_{0}\rho RT/\left(b{}_{K}GM{}_{0}\right)}$ , where *b*_K_ is the polymer Kuhn length (here, *b*_K_=0.8 nm is used) enables the calculation of *ϵ*_max_, the maximum theoretical extension at break as *ϵ*_max_=(*λ*_max_−1)×100 %.

The remarkable mechanical properties of our materials can further be assessed in the nonlinear deformation regime (Figure 3, Table 1). Under this regime, the materials with 80 % *cis* isomer content are strong and elastic materials with an ultimate tensile strength (UTS) of 54.3±6.5 MPa and an elongation at break (*ϵ*_break_) of 1495±66 % (Figure 3 A). The degree of control over network elastic properties by varying the *cis* isomer content, is clearly illustrated from samples with 52 % and 32 % *cis* isomer content, respectively (Figures 3 B and C). In these examples, increasing the fraction of *cis* isomers leads to a decrease of the elongation at break from 2970±137 % to 2252±115 %. This insignificant decrease in network elongation is compensated by almost a five‐fold increase from 2.82±0.4 MPa to 16.6±3.8 MPa in ultimate tensile strength at break (Table 1). The integral characteristic of ultimate tensile strength and elongation at break is material's tensile toughness. For these materials, it varies between 35±3 MJ m^−3^ and 330±29 MJ m^−3^, which indicates that a large amount of energy can be stored in the system either by achieving a high ultimate tensile strength or elongation at break (see data for 70 % and 80 % *cis* isomer content). The values obtained are noted to differ from the measured elongation at break, *ϵ*_break_ (Table 1) as a consequence of elongation induced crystallization of the samples and sample necking at large deformations which is not captured in the *ϵ*_max_ estimated from small network deformations. Nonetheless, this is consistent with what is expected for amorphous entangled networks with shear modulus values on the order of 1–5 MPa.^8^ Finally, a series of experiments were conducted to investigate the effect of molar mass of precursor polymers on the mechanical properties of resultant networks. The preparation of a series of samples with *M*_w_=44, 62 and 107 kDa and subsequent analysis revealed a decrease in ultimate tensile strength (UTS*)* and a slight increase in the Young's modulus *E* with decreasing molar mass (Supporting Information, Table S3). These changes are likely a result of more facile and rapid crystallization of the lower molar mass materials.^9^

**Figure 3:**
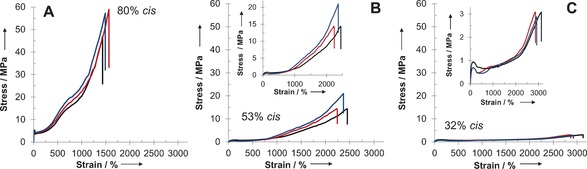
A)–C) Exemplary stress vs. strain curves for A) 80 % *cis* C_3A_–C_6S_; B) 53 % *cis* C_3A_–C_6S_; C) 32 % *cis* C_3A_–C_6S_ in the non‐linear region. Data for three samples are shown to illustrate the reproducibility. Expansions inset for clarity.

The synthetic nature of this system also affords the ability to control material properties via monomer design and end‐group modification. This method results in controllable and definable end groups that can be specifically modified and will be critical to dispersing and grafting fillers within the elastomer network. Specifically, the use of a slight excess of dialkyne monomer results in alkyne chain ends that can be further modified by a simple nucleophilic addition or click reaction. To this end, we have demonstrated that 2,2,2‐trifluoroethanethiol can be added selectively to the chain ends of C_3A_–C_6S_ (Figure 4) by addition of the thiol to the material either directly after the polymerization reaction or in a subsequent post‐polymerization step. The two signals are consistent with *cis* and *trans* double‐bond formation upon addition. This has been confirmed by ^1^H and ^19^F NMR spectroscopy of small molecule model compounds (data not shown). Beyond chain‐end modification, simple variation of the monomer structure of the dialkyne or dithiol enables not only access to varied mechanical properties but also materials with different functional end groups. For example, materials with longer alkyl chains between ester units (introduced through either dialkyne or dithiol monomers) typically resulted in materials with increased strength (Supporting Information, Table S4). C_3A_–C_3S_ materials were also prepared and characterized and confirmed that the manipulation of *cis*/*trans* ratio retained the effect on mechanical properties for a different monomer composition (Supporting Information, Table S4). Distinct changes in physical properties were easily introduced through the incorporation of functional dithiols including 2,2′‐(ethylenedioxy)diethanethiol and 1,4‐dithio‐d‐threitol. Even at only a 10 % incorporation of these comonomers, the polar component of the surface energy is significantly reduced, while the mechanical properties were largely retained (Supporting Information, Tables S4 and S5). These effects will be significant when dispersing polar filler materials.

**Figure 4:**
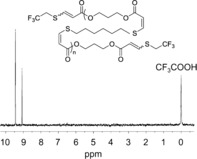
^19^F NMR spectrum of C_3A_–C_6S_ thiol–yne step‐growth polymer following end capping with 2,2,2‐trifluoroethanethiol (376 MHz, CDCl_3_+0.01 % v/v CF_3_COOH).

Analysis of the hydrolytic and thermal degradability of the materials have also been investigated. Storage at ambient temperature leads to no observable discoloration or change in mechanical properties. Submersion of the 80 % *cis* C_3A_–C_6S_ material in both phosphate‐buffered saline solution (pH 7.4) and 1 m KOH_aq_ solution at 27 °C only revealed less than 0.3 % addition of mass and no degradation after 14 days, which indicates that the materials have an excellent stability to hydrolysis. Furthermore, using thermogravimetric analysis (TGA), an onset of degradation was observed at 355 °C which shows that the materials are also thermally stable and suitable for melt processing, thermal molding and vulcanization‐ based toughening. These materials are shown to be stable to ambient oxidation and maintained their mechanical properties for more than a year. Finally, we have demonstrated that the double bond within the material also remains accessible for further reaction. Specifically, vulcanization of the materials by radical curing with 1 wt % dicumyl peroxide at 160 °C in bulk yields a significant change to the materials properties such that, consistent with cross‐linking reactions occurring, the material becomes more elastic in nature (Figure 5). Most notably, the modified material can now be subjected to repeated load–unload cycles up to 500 % extension before being allowed to relax. While the non‐vulcanized sample does not significantly recover, the vulcanized sample recovers to 150 % of its original length, which demonstrates the superior elastic properties endowed as a result of the vulcanization process.

**Figure 5:**
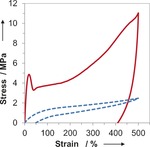
Exemplary stress vs. strain curves for 80 % *cis* C_3A_‐C_6S_ before (red solid line) and after (blue dashed line) vulcanization with 1 %wt dicumyl peroxide.

The novel approach to materials design described herein outlines a series of principles that transfer the unique mechanical properties of natural rubber and gutta percha to a fully synthetic system. The organocatalytic step‐growth polymerization affords independent control over mass, mass distribution, stereochemistry, and the resulting mechanical and physical properties, without the use of metals or exotic additives. Furthermore, the synthetic precursors allow access to starting materials and functional species and defined end groups not commonly found in natural or even synthetic elastomers. The benefits of being able to control monomer composition, stoichiometry, end‐group modification with a minimal resulting effect on mechanical performance combined with the ability to toughen via commercially relevant vulcanization processes yields an exciting design space for functional elastomers.

## Supporting information

As a service to our authors and readers, this journal provides supporting information supplied by the authors. Such materials are peer reviewed and may be re‐organized for online delivery, but are not copy‐edited or typeset. Technical support issues arising from supporting information (other than missing files) should be addressed to the authors.

Supplementary
