# Supplementary material for: Independent Control of Elastomer Properties through Stereocontrolled Synthesis
Source: Angew Chem Int Ed Engl. 2016 Oct 6;55(42):13076–80. doi: 10.1002/anie.201606750 (PMC5082520; doi:10.1002/anie.201606750)
Supplement: Supplementary file 1 — Supplementary [file ANIE-55-13076-s001.pdf]

Angewandte Chemie
Eine Zeitschrift der Gesellschaft Deutscher Chemiker

Supporting Information

Independent Control of Elastomer Properties through Stereocontrolled Synthesis

Craig A. Bell[+], Jiayi Yu[+], Ian A. Barker, Vinh X. Truong, Zhen Cao, Andrey V. Dobrinyin, Matthew L. Becker, and Andrew P. Dove**

anie_201606750_sm_miscellaneous_information.pdf

This PDF file includes:

Materials and Methods
Supplementary Text
Figs. S1 to S18
Tables S1 to S6

Materials and Methods

Materials

Reagents

The following chemicals were used as received: acetonitrile (MeCN: Sigma-Aldrich, > 99.5%), 1,4-butanediol (Sigma-Aldrich, 99%), chloroform ($CHCl_3$: VWR Chemicals, 99%), *d*-chloroform ($CDCl_3$: Apollo, > 99%), 1,8-diazabicyclo[5.4.0]undec-7-ene (DBU: Sigma-Aldrich, 98%), diethyl ether (Et_2O: Sigma-Aldrich, ≥ 99.8%), *N*,*N*-dimethylformamide (DMF: Fisher Scientific, LR grade), 2,6-di-*tert*-butyl-4-methylphenol (BHT: Alfa Aesar, 99%), ethyl acetate (EtOAc: Fisher Scientific, LR grade), hexane (VWR Chemicals, 99%), 1,6-hexanediol (Sigma-Aldrich, 99%), magnesium sulfate ($MgSO_4$: anhydrous, Fisher Scientific, LR grade), 1,3-propanediol (Sigma-Aldrich, 98%), propiolic acid (Acros Organics, 98%), silica gel (SiO_2: Apollo Scientific, 40-63 micron), sodium chloride (NaCl: Fisher Scientific, > 99%), sodium hydrogen carbonate ($NaHCO_3$: Fisher Scientific, > 99%), sulfuric acid (Fisher Scientific, > 95%), triethylamine (Et_3N: Fisher Scientific, LR grade), trifluoroacetic acid (CF_3COOH: Sigma-Aldrich, 99%).

The following chemicals were vacuum distilled prior to use and stored in Young's tapped ampoules under N_2: 1,4-butanedithiol (Sigma-Aldrich, 97%), 1,10-decanedithiol (Alfa Aesar, 95%), 2,2′-(ethylenedioxy)diethanethiol (DEGDT: Sigma-Aldrich, 95%), 1,6-hexanedithiol (Sigma-Aldrich, ≥ 97%), 1,8-octanedithiol (Alfa Aesar, 98%), 1,3-propanedithiol (Sigma-Aldrich, 99%), 2,2,2-trifluoroethanethiol (Sigma-Aldrich, 95%). 1,4-dithio-*D*-threitol (DTT: Sigma-Aldrich, ≥ 98%) was purified by sublimation under high vacuum prior to use and stored at -20 °C.

Methods

Synthetic Procedures

Propane-1,3-diyl dipropiolate, 1. 1,3-Propanediol (20 g, 0.263 mol) was added to a 1 L single neck round bottom flask. To this was added toluene (100 mL) and benzene (100 mL). Two drops of H_2SO_4 were added and the solution was allowed to stir at room temperature for 5 min before adding propiolic acid (50 g, 0.714 mol). A Dean-Stark apparatus with condenser was fitted and the reaction was then refluxed for 16 h at 120 °C or until the required amount of water was collected. The solution was then cooled to room temperature and solvent extracted with saturated $NaHCO_3$ solution (2 × 200 mL) to remove any residual acids. The organic phase was then collected, dried over $MgSO_4$, filtered and reduced in volume to dryness. The product was then purified on silica gel isocratically using 4:1 hexane/EtOAc and collecting the 1st fraction. After removal of the solvent, the final product was further purified by distillation under high vacuum at 160 °C to yield colourless oil that slightly crystallised on sitting (24.63 g, 52% yield). R_f (3:2 Hex/EtOAc) = 0.43. Melting point: 25 °C. ^{1}H NMR (500 MHz, $CDCl_3$) δ 4.30 (t, $^3J_{HH}$ = 6.2 Hz, 4H), 2.88 (s, 2H), 2.19 – 1.96 (m, 2H). ^{13}C NMR (125 MHz, $CDCl_3$) δ 152.6, 75.3, 74.5, 62.6, 27.5. ESI-MS Calcd for $C_9H_8O_4Na$ (M+Na): 203.03, Found: 203.0. Anal Calcd for $C_9H_8O_4$: C 60.00; H 4.48 %. Found: C 59.70; H 4.41 %.

Butane-1,4-diyl dipropiolate, 2. Synthetic procedure was identical to **1**. The final product was further purified by sublimation under high vacuum at 50 °C to yield

clear/white crystals (26.88 g, 83% yield). R_f (3:2 Hex/EtOAc) = 0.44. Melting point: 50 °C. ^{1}H NMR (500 MHz, $CDCl_3$) δ 4.23 (t, $^3J_{HH}$ = 6.5 Hz, 4H), 2.89 (s, 2H), 1.90 – 1.67 (m, 4H). ^{13}C NMR (125 MHz, $CDCl_3$) δ 152.8, 75.0, 74.7, 65.6, 25.0. ESI-MS Calcd for $C_{10}H_{10}O_4Na$ (M+Na): 217.05, Found: 216.9. Anal Calcd for $C_{10}H_{10}O_4$: C 61.85; H 5.19 %. Found: C 61.89; H 5.13 %.

Hexane-1,6-diyl dipropiolate, 3. Synthetic procedure was identical to **1**. The final product was further purified by recrystallization onto a cold finger under high vacuum at 75 °C to yield clear/white crystals (13.58 g, 48% yield). R_f (3:2 Hex/EtOAc) = 0.49. Melting point: 53 °C. ^{1}H NMR ($CDCl_3$, 500 MHz) δ 4.19 (t, $^3J_{HH}$ = 6.6 Hz, 4H), 2.88 (s, 2H), 1.76 – 1.62 (m, 4H), 1.48 – 1.34 (m, 4H). ^{13}C NMR (125 MHz, $CDCl_3$) δ 152.9, 74.8, 74.7, 66.3, 28.3, 25.5. ESI-MS Calcd for $C_{10}H_{10}O_4Na$ (M+Na): 245.08, Found: 244.9. Anal Calcd for $C_{12}H_{14}O_4$: C 64.85; H 6.35 %. Found: C 64.68; H 6.32 %.

Synthesis of propane-1,3-diyl (2E,2'E)-bis(3-(butylthio)acrylate), 4. Propane-1,3-diyl dipropiolate (213 mg, 1.2 mmol) in $CHCl_3$ (2 mL) was added drop-wise to a solution of 1-butanethiol (257 µL, 2.4 mmol) and Et_3N (1.7 µL, 0.012 mmol) in $CHCl_3$ (4 mL) at 0 °C with stirring. After full addition the solution was then allowed to stir at ambient temperature for 120 min, after which the solution was solvent extracted with 1M HCl (2 × 20 mL) and saturated NaCl solution (20 mL). The organic phase was dried over $MgSO_4$, filtered and taken to dryness to yield a colourless oily product (358 mg, 84% yield). R_f (3:2 Hex/EtOAc) = 0.53. ^{1}H NMR (500 MHz, $CDCl_3$) δ 7.69 (d, $^3J_{HH}$ = 15.1 Hz, 1H), 5.72 (d, $^3J_{HH}$ = 15.1 Hz, 1H), 4.22 (t, $^3J_{HH}$ = 6.3 Hz, 2H), 2.79 (t, $^3J_{HH}$ = 7.4 Hz, 2H), 2.08 – 1.96 (m, 2H), 1.72 – 1.62 (m, 4H), 1.51 – 1.37 (m, 4H), 0.94 (t, $^3J_{HH}$ = 7.4 Hz, 6H). ^{13}C NMR (125 MHz, $CDCl_3$) δ 165.4, 147.6, 113.3, 61.1, 31.8, 30.7, 28.3, 22.1, 13.7. ESI-MS Calcd for $C_{17}H_{28}O_4S_2Na^+$ (M-Na^+): 383.13, Found: 383.1. Anal Calcd for $C_{17}H_{28}O_4S_2$: C 56.64; H 7.83 %. Found: C 56.65; H 7.90 %.

Synthesis of propane-1,3-diyl (2Z,2'Z)-bis(3-(butylthio)acrylate), 5. Propane-1,3-diyl dipropiolate (216 mg, 1.2 mmol) in MeCN (2 mL) was added drop-wise to a solution of 1-butanethiol (257 µL, 2.4 mmol) and DBU (1.8 µL, 0.012 mmol) in MeCN (4 mL) at 0 °C with stirring. After full addition the solution was then allowed to stir at ambient temperature for 30 min, after which the MeCN was evaporated *in vacuo*. The resultant solid was recrystallised from hexane 2 times to yield the product as white crystals (242 mg, 56% yield). R_f (3:2 Hex/EtOAc) = 0.53. Melting point: 51 °C. ^{1}H NMR (500 MHz, $CDCl_3$) ^{1}H NMR (500 MHz, $CDCl_3$) δ 7.09 (d, $^3J_{HH}$ = 10.2 Hz, 1H), 5.82 (d, $^3J_{HH}$ = 10.2 Hz, 1H), 4.24 (t, $^3J_{HH}$ = 6.3 Hz, 2H), 2.75 (t, $^3J_{HH}$ = 7.4 Hz, 2H), 2.11 – 1.92 (m, 2H), 1.73 – 1.54 (m, 4H), 1.52 – 1.31 (m, 4H), 0.92 (t, $^3J_{HH}$ = 7.4 Hz, 6H). ^{13}C NMR (125 MHz, $CDCl_3$) δ 166.7, 150.9, 112.8, 60.9, 35.9, 32.5, 28.2, 21.7, 13.7. ESI-MS Calcd for $C_{17}H_{28}O_4S_2Na^+$ (M-Na^+): 383.13, Found: 383.1. Anal Calcd for $C_{17}H_{28}O_4S_2$: C 56.64; H 7.83 %. Found: C 56.68; H 7.89 %.

Synthesis of propane-1,3-diyl bis(3-((2,2,2-trifluoroethyl)thio)acrylate), 6. Propane-1,3-diyl dipropiolate (215 mg, 1.2 mmol) in $CHCl_3$ (2 mL) was added drop-wise to a solution of 2,2,2-trifluoroethanethiol (214 µL, 2.4 mmol) and Et_3N (1.7 µL, 0.012 mmol) in $CHCl_3$ (4 mL) at 0 °C with stirring. After full addition the solution was then

allowed to stir at ambient temperature for 120 min, after which the solution was solvent extracted with 1 M HCl (2 × 20 mL) and saturated NaCl solution (20 mL). The organic phase was dried over $MgSO_4$, filtered and taken to dryness to yield a colourless oily product that crystallised on standing. The solid was recrystallised twice from hexanes/EtOAc (19:1) to yield white crystals (472 mg, 96% yield). R_f (3:2 Hex/EtOAc) = 0.38. Melting points: -40, 58 and 79 °C (mixture of 3 isomers E,E', E,Z', and Z,Z'). ^{1}H NMR (500 MHz, $CDCl_3$) δ 7.56 (d, $^3J_{HH}$ = 15.3 Hz, 1H), 7.02 (d, $^3J_{HH}$ = 8.0 Hz, 1H), 5.94 (dd, $^3J_{HH}$ = 12.6 Hz, $^2J_{HH}$ = 9.7 Hz, 2H), 4.42 – 4.07 (m, 4H), 3.52 – 3.14 (m, 4H), 2.19 – 1.88 (m, 2H). ^{13}C NMR (125 MHz, $CDCl_3$) δ 166.4, 164.6, 147.8, 147.8, 144.3, 144.2, 128.4, 128.1, 126.2, 125.9, 124.0, 123.7, 121.8, 121.5, 116.75, 116.7, 115.2, 115.1, 61.4, 61.3, 61.3, 38.4, 38.1, 37.85, 37.6, 35.5, 35.3, 35.0, 34.7, 28.1, 28.1, 28.1. ^{19}F NMR (376 MHz, $CDCl_3$ + 0.01% v/v CF_3COOH) δ 9.44, 9.08, 0.00 (CF_3COOH). ESI-MS Calcd for $C_{13}H_{14}F_6O_4S_2Na^+$ (M-Na^+): 435.01, Found: 435.0. Anal Calcd for $C_{13}H_{14}F_6O_4S_2$: C 37.87; H 3.42 %. Found: C 37.83; H 3.31 %.

General procedure for thiol-yne step growth polymerisation. An example of the thiol-yne step growth polymerisation is as follows: 1,3-propanedithiol (3.24 g, 2.99 × 10^{-2} mol) was added to a 100 mL round bottom flask. To this propane-1,3-diyl dipropiolate (5.40 g, 3.00 × 10^{-2} mol) was added by quantitative transfer with $CHCl_3$ (60 mL). The solution was then cooled to -15 °C with stirring for 15 min before DBU (45 μL, 3.00 × 10^{-4} mol) was added in one portion. The addition of DBU produced an exotherm causing the solvent to bubble. After 2 min of stirring, the reaction was then allowed to warm to room temperature and continued to stir, during which time the solution became very viscous. After 1 h the solution was diluted with $CHCl_3$ (40 mL) and BHT (0.5 g, mol) was added in order to prevent crosslinking or further reaction during the precipitation steps. The polymer solution was then precipitated into diethyl ether (1000 mL) and collected by decanting the supernatant. The polymer was then redissolved in $CHCl_3$ (100 mL) and reprecipitated into diethyl ether (1000 mL), collected by decanting the supernatant, and dried *in vacuo* at room temperature for 24 h. SEC ($CHCl_3$ + 0.5% Et_3N) M_n = 29.4 kDa, M_w = 151.3 kDa, M_p = 155.3 kDa, $Đ_M$ = 5.14. ^{1}H NMR ($CDCl_3$, 400 MHz) % *cis* = 75%.

Variation of molecular weight. The molecular weight of the thiol-yne step growth polymers was varied by changing the amount of dithiol in relation to the dialkyne such that the dialkyne was always in excess. Monomer ratios were determined using the extended Carothers equation for one monomer in excess (assuming $p \rightarrow 100\%$).[1]

Variation of % cis. The % *cis* in each thiol-yne step growth polymer was tuned based on solvent polarity and base. Truong and Dove[2] have shown that low and high % *cis* can be achieved by changing the base from Et_3N to DBU while maintaining the solvent ($CDCl_3$). However, moderately high % *cis* can be achieved with Et_3N when a more polar solvent such as DMSO is used. All high % *cis* polymers were formed using DBU/$CHCl_3$ but lower % *cis* contents were formed by using Et_3N and varying compositions of $CHCl_3$ and DMF (17:3, 7:3, and 100% DMF). The double bond stereochemistry was determined by integration of the resonances in the 1H NMR spectrum that are attributed to *cis* and *trans* double bond isomers respectively. These

signals typically appeared at *ca*. δ = 5.9 and 7.1 ppm for the *cis* isomer and δ = 5.8 and 7.6 ppm for the *trans* isomer (in $CDCl_3$). Critically, they can be distinguished from their different coupling constants (*cis*: $^3J_{H\text{-}H}$ = 10.2 Hz, *trans*: $^3J_{H\text{-}H}$ = 15.2 Hz).

Chain end modification of C_{3A}-C_{6S} polymer. To 100 mg of C_{3A}-C_{6S} thiol-yne step growth polymer (M_w = 132.4 kDa, $Đ_M$ = 7.25) was added $CHCl_3$ (5 mL) and 2,2,2-trifluoroethanethiol (2.4 μL, 2.7 × 10^{-5} mol). This was allowed to stir until completely dissolved before adding Et_3N (1 μL, 7.2 × 10^{-6} mol). The solution was stirred at room temperature for 16 h before precipitating into diethyl ether. The polymer was collected and redissolved in $CHCl_3$ before reprecipitating into diethyl ether. Upon collection the polymer was dried *in vacuo* at room temperature for 24 h.

General Method for Radical Curing. 2 g of C_{3A}-C_{6S} polymer was dissolved in CH_2Cl_2 (20 mL) and to this was added 1 wt% of dicumyl peroxide (20 mg). The solution was stirred overnight then reduced to dryness *in vacuo*. The polymer was placed into a mould and cured at 160 °C for 15 min before being pressed into films.

NMR Spectroscopy

NMR (^{1}H, ^{13}C, and ^{19}F) spectra were recorded in $CDCl_3$ on either a Bruker DPX-500 (monomers and model compounds) or Bruker DPX-400 (all polymers) spectrometer at 298 K. Chemical shifts are reported as δ in parts per million (ppm) and referenced to the chemical shift of the residual solvent resonances ($CDCl_3$ ^{1}H: δ = 7.26 ppm, ^{13}C: δ = 77.16 ppm) or to external standards (CF_3COOH ^{19}F: δ = 0 ppm). The resonance multiplicities are described as s (singlet), d (doublet), t (triplet), q (quartet) or m (multiplet).

Size-exclusion Chromatography (SEC)

SEC was used to determine the molar masses and molar mass distributions (dispersities, $Đ_M$) of the synthesised polymers. SEC analyses were performed on a system composed of an Agilent 390-LC-Multi detector using an Agilent Polymer Laboratories guard column (PLGel 5 μM, 50 × 7.5 mm), two mixed D Varian Polymer Laboratories columns (PLGel 5μM, 300 × 7.5 mm) and a PLAST RT autosampler. Detection was conducted using a differential refractive index (RI) and an ultraviolet (UV) detector set to 280 nm. The analyses were performed in $CHCl_3$ at 40 °C and containing 0.5% w/w Et_3N at a flow rate of 1.0 mL/min. Polystyrene (PS) (162 – 2.4 × 105 g.mol^{-1}) standards were used to calibrate the system. Polymers containing 2,2′-(ethylenedioxy)diethanethiol and 1,4-dithio-*d*-threitol were analysed on a system composed of an Agilent 390-LC-Multi detector using an Agilent Polymer Laboratories guard column (PLGel 5 μM, 50 × 7.5 mm), two mixed C Varian Polymer Laboratories columns (PLGel 5μM, 300 × 7.5 mm) and a PLAST RT autosampler. Detection was conducted using a differential refractive index (RI). The analyses were performed in DMF at 50 °C and containing 5 mM NH_4BF_4 at a flow rate of 1.0 mL/min. Poly(methyl methacrylate) (PMMA) (550 – 2.136 × 10^6 g.mol^{-1}) standards were used to calibrate the system. Molecular weights and dispersities were determined using Cirrus v2.2 SEC software. Absolute MW size exclusion chromatography (SEC) analysis was performed on an Agilent 390-LC MDS

instrument equipped with differential refractive index (DRI), viscometry (VS), dual angle light scatter (LS) and two wavelength UV detectors. The system was equipped with 2 × PLgel Mixed D columns (300 × 7.5 mm) and a PLgel 5 µm guard column. The eluent used was $CHCl_3$ with 2% Et_3N. Samples were run at 1 $mL.min^{-1}$ at 30 °C. Poly(methyl methacrylate), and polystyrene standards (Agilent EasyVials) were used for calibration and ethanol was added as a flow rate marker. Analyte samples were filtered through a PTFE membrane with 0.22 µm pore size before injection.

Thermal Property Measurements

The thermal characteristics of the polymers were determined using differential scanning calorimetry (DSC, TA Q200) from -50 °C to 150 °C at a scanning rate of 10 °C/min. The glass transition temperature (T_g) was determined from the midpoint in the second heating cycle of DSC.

Mechanical Property Measurements

Tensile Tests at Different Strain Rates

Thin films of each polymer were fabricated using a vacuum compression machine (TMP Technical Machine Products Corp.). The machine was preheated to 160 °C. Then polymer was added into the 50 × 50 × 0.5 mm mould and put into the compression machine with vacuum on. After 15 minutes of melting, the system was degassed three times. Next, 10 lbs*1000, 15 lbs*1000, 20 lbs*1000, 25 lbs*1000 of pressure were applied for 2 minutes respectively. After that, the mould was cooled down with 1000 psi of pressure to prevent the wrinkle on the film's surface. The films were visually inspected to ensure that no bubbles were present in the films. Dumbbell-shaped samples were cut using a custom ASTM Die D-638 Type V.

Tensile tests at different stretching speed were carried out using Instron (Instron 5543 Universal Testing Machine) at room temperature (25 ± 1 °C). The gauge length was set as 7 mm and the crosshead speed was set as various values (listed in Table S1). The dimensions of the neck of the specimens were 7.11 mm in length, 1.70 mm in width and 0.50 mm in thickness.

Tensile Tests at 20 mm/min

Dumbbell-shaped samples were prepared using the same method as stated previously. Tensile tests were carried out using Instron (Instron 5543 Universal Testing Machine) at room temperature (25 ± 1 °C). The gauge length was set as 7 mm and the crosshead speed was set as 20 mm/min. The dimensions of the neck of the specimens were 7.11 mm in length, 1.70 mm in width and 0.50 mm in thickness. The elastic moduli were calculated using the slope of linear fitting of the data from strain of 0% to 0.1%. The reported results are average values from three individual measurements.

Wide Angle X-ray Diffraction Measurement (WAXD).

The WAXD experiments were conducted using a Rigaku Ultima IV X-ray Diffractometer at room temperature. The generator was operated at 40 kV and 40 mA with a beam monochromatized to Cu Kα radiation. Samples were scanned at 1.0 degree/min continuously over a scan range of 5.0 - 70.0 degree. The air scattering was subtracted.

Small Angle X-ray Scattering Measurement (SAXS).

The SAXS experiments were performed on a Rigaku MicroMax 002+ instrument equipped with a two-dimensional multiwire area detector and a microfocus sealed copper tube. The working voltage and current used were 45 kV and 0.88 mA, with the wavelength of the X-ray is 0.154 nm. The scattering vector (q) was calibrated using standard of silver behenate with the primary reflection peak at (q = 1.067 nm^{-1}). The recording time for each sample was 10−20 min, depending on the scattering intensity. The data analysis was done with SAXSgui software.

Network Mechanical Properties

According to Boltzmann superposition principle,[3] we can describe stress evolution in polymeric networks undergoing uniaxial elongation at a constant strain rate $\dot{\varepsilon}$ as

$$\sigma(t) = \int_0^t E(t-t')d\varepsilon(t') = \int_0^t E(t-t')dt' = \dot{\varepsilon}\int_0^t E(\Delta t)d\Delta t \quad \text{(S1)}$$

where $E(t)$ is time dependent network Young's modulus. In entangled amorphous polymeric networks, dynamics of entangled strands forming a network on the time scales t smaller than the strands' Rouse time,$\tau_R = \tau_0 N_e^2$ (N_e - degree of polymerization of network strands between entanglements and τ_0 - characteristic monomer time), the dynamics of network strands is not influenced by entanglements or crosslinks and network modulus decays with time as, $E(t) \propto \rho kT(\frac{\tau_0}{t})^{1/2}$, where ρ is a monomer number density, k is the Boltzmann constant and T is the absolute temperature. However, at the time scales t larger than the Rouse time of the entangled polymeric strands the network response is pure elastic with Young's modulus $E \propto \rho kT/N_e$. We will use the following approximation for time dependent modulus to describe network relaxation:

$$E(t) \approx 3\rho kT \begin{cases} (\tau_0/t)^{1/2}, & for \quad \tau_0 < t \le \tau_R = \tau_0 N_e^2 \\ 1/N_e, & for \quad \tau_R \le t \end{cases} \quad \text{(S2)}$$

For the stress relaxation modulus given by eq S2 integration of eq S1 results in

$$\sigma(t) \approx 2E_m \dot{\varepsilon}\tau_0^{1/2} t^{1/2}, \qquad for\ t < \tau_R \quad \text{(S3)}$$

where we introduced $E_m = 3\ kT/v$ with monomer volume v. For experiments at constant strain rate, we can substitute $t = \varepsilon/\dot{\varepsilon}$ into eq S3 to express $\sigma(t)$ as a function of strain ε as

$$\sigma(\varepsilon) \approx 2E_m \tau_0^{1/2}\dot{\varepsilon}^{1/2}\varepsilon^{1/2}, \quad for\ t < \tau_R \quad \text{(S4)}$$

In the opposite limit, $t > \tau_R$,

$$\sigma(t) \approx E_m \dot{\varepsilon} t/N_e, \qquad for\ t \ge \tau_R \quad \text{(S5)}$$

Analysis of the eq S5 shows that in this network deformation regime we can collapse data sets obtained at different strain rates by plotting $\frac{\sigma(t)}{\dot{\varepsilon}}$ vs $\varepsilon/\dot{\varepsilon}$. The slope will provide value

of the network Young's modulus at small deformations, $E_0 = E_m/N_e$. Note, that in the case of composite materials with a spectrum of relaxation times, eq S5 is valid on the time scales longer than the longest relaxation time of stress relaxation in a system.

Network tensile toughness reported in Table I was calculated from the area under the stress-strain curve

$$U_{TT} = \int_0^{\varepsilon_{break}} \sigma d\varepsilon \qquad \text{(S6)}$$

where ε_{break} is the strain upon failure.

Surface Energy Measurement.

The surface energies of the polymers were estimated from contact angle measurements. By measuring contact angle with four different probe liquids (propylene glycol, ethylene glycol, glycerol and water) using an Advanced Goniometer (Rame-Hart Instrument Co., Model 500) at 25 °C and fitting the data using the Owen's equation, the dispersion and polar components of the polymer surface energy before stretching and after stretching were obtained from the slope and the intercept (Table S6).[4-6] At least five measurements were performed on different spots and averaged.

Owen's equation: $$\frac{(1+cos\theta_{LP})\gamma_L}{2\sqrt{\gamma_L^d}} = \sqrt{\gamma_S^d} + \sqrt{\frac{\gamma_L^p}{\gamma_L^d}}\sqrt{\gamma_S^p}$$

$$\gamma_S = (\sqrt{\gamma_S^d})^2 + (\sqrt{\gamma_S^p})^2$$

where θ_{LP} is the Young's contact angle, γ_L is the liquid surface tension, γ_L^d is the dispersion component of liquid surface tension, γ_L^p is the polar component of liquid surface tension, γ_L is the polymer surface energy, γ_S^d is the dispersion component of polymer surface energy and γ_S^p is the polar component of polymer surface energy.

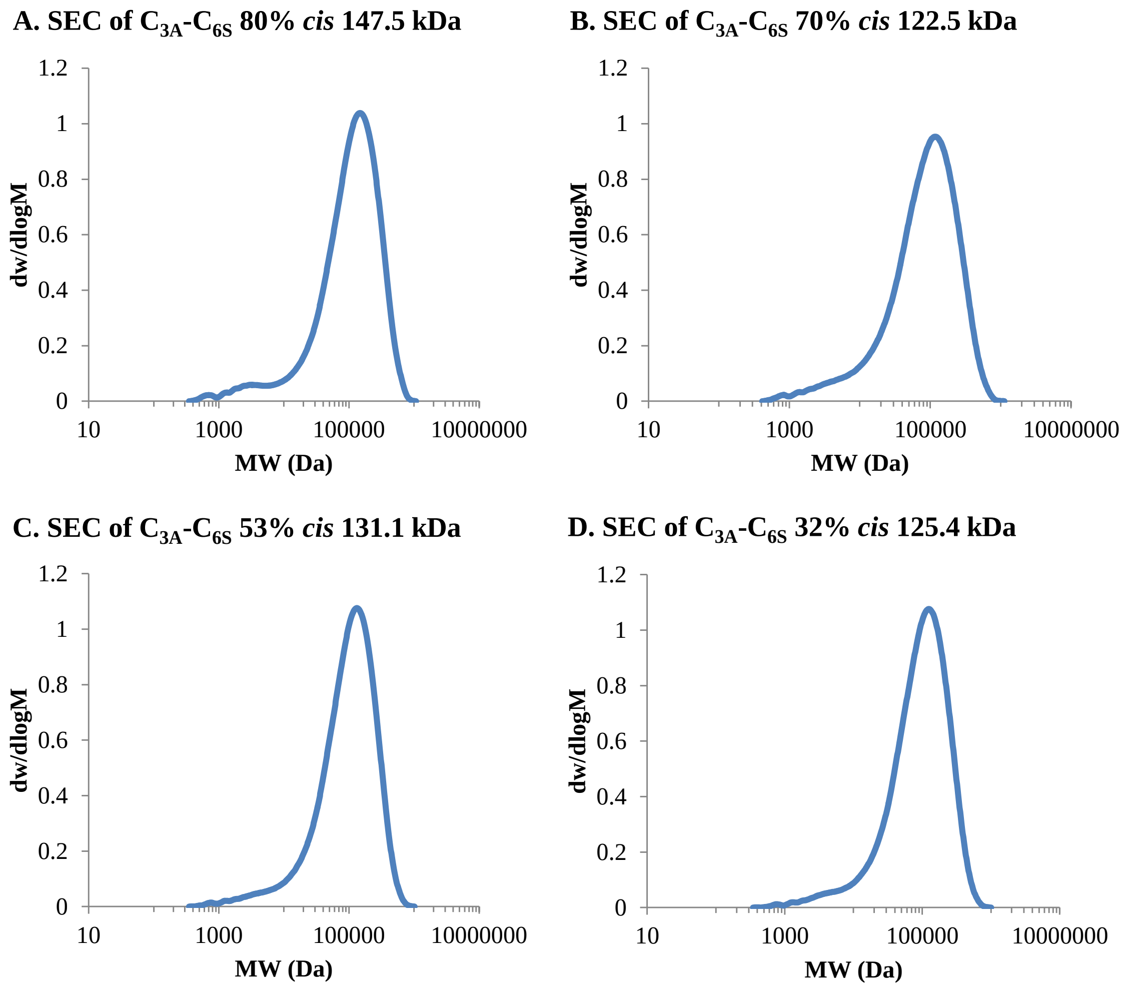

Fig. S1.

SEC chromatograms of C_{3A}-C_{6S} polymers with different %*cis* double bonds (80, 70, 53, 32%). Molecular weights given are the weight average molecular weight (M_w).

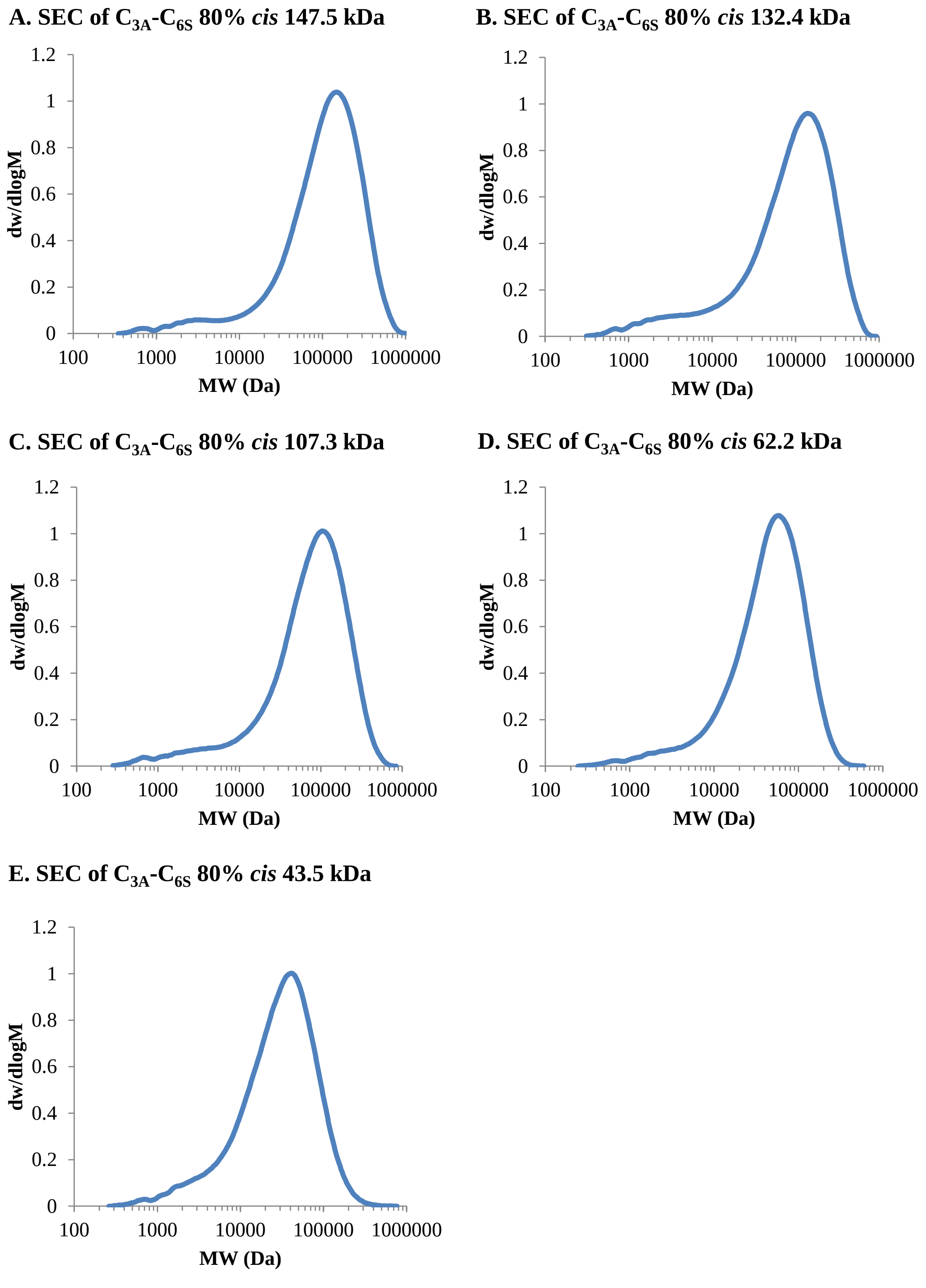

Fig. S2.

SEC chromatograms of C_{3A}-C_{6S} polymers containing 80% *cis* double bonds at different molecular weights (147.5, 132.4, 107.3, 62.2, and 43.5 kDa). Molecular weights given are the weight average molecular weight (M_w).

A. ^{1}H NMR spectrum of C$_{3A}$-C$_{6S}$, 80% *cis*

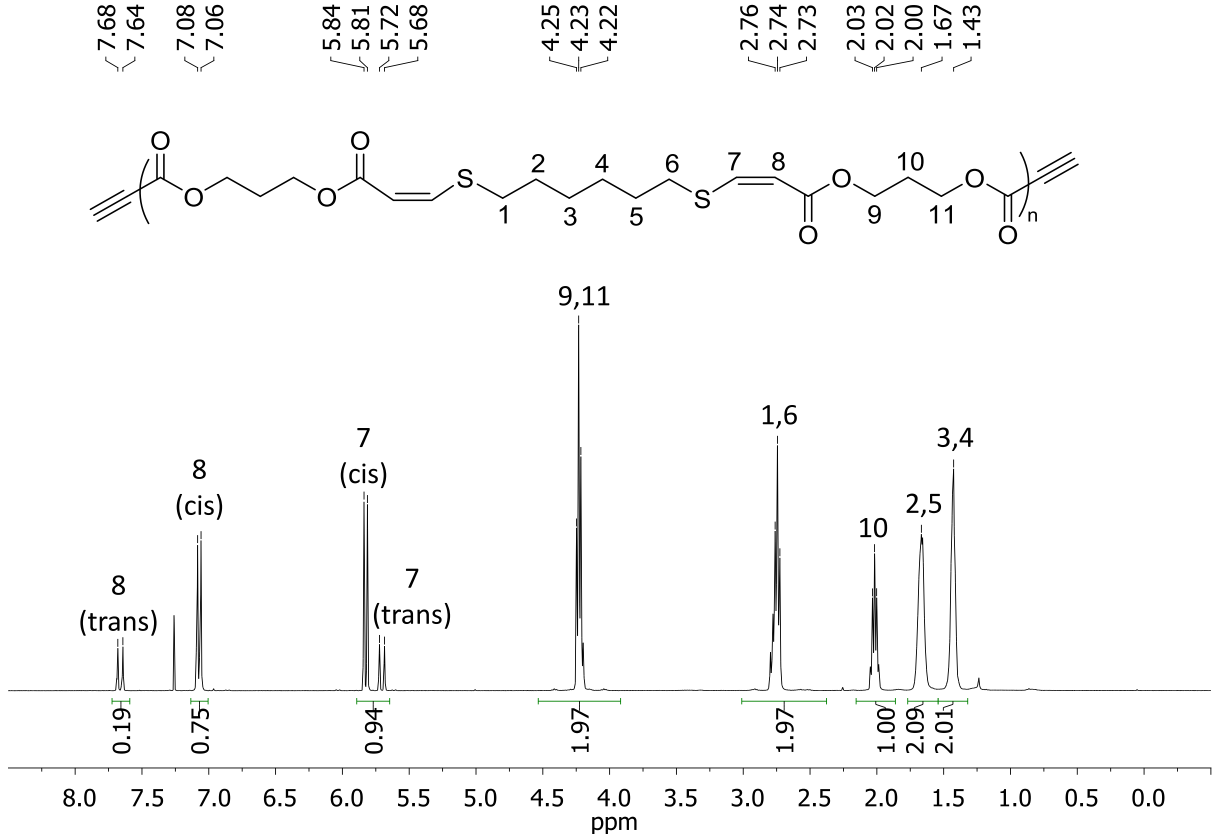

B. ^{1}H NMR spectrum of C$_{3A}$-C$_{6S}$, 70% *cis*

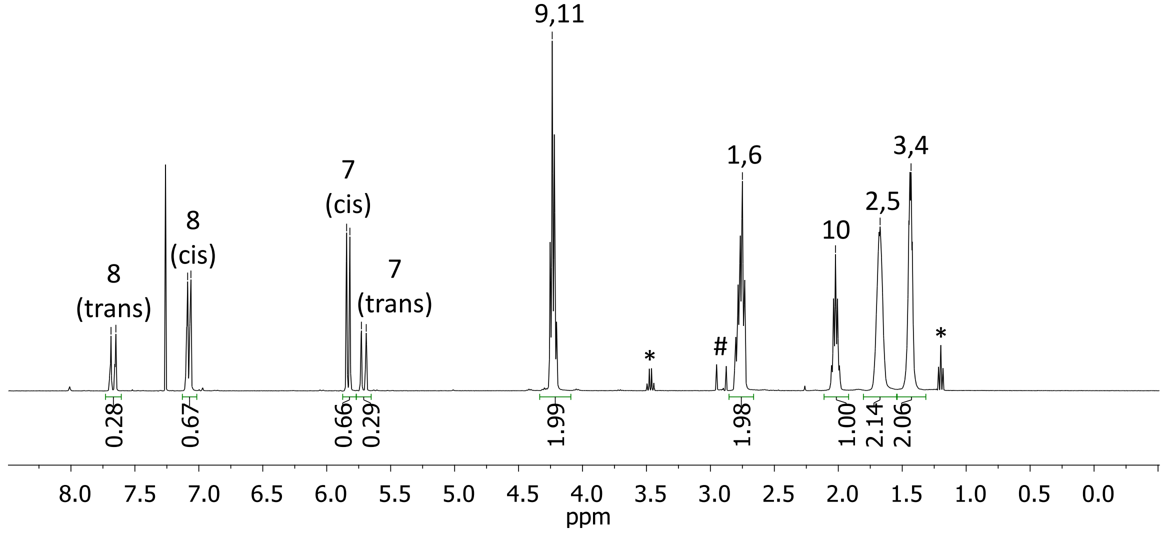

C. ^{1}H NMR spectrum of C3A-C6S, 53% *cis*

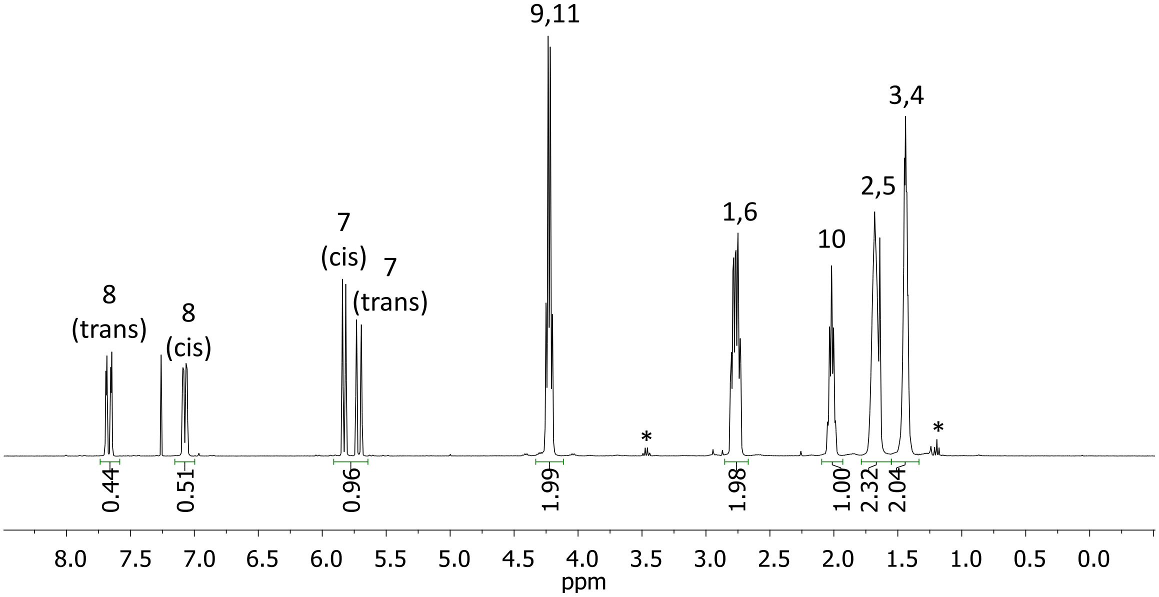

D. ^{1}H NMR spectrum of C3A-C6S, 32% *cis*

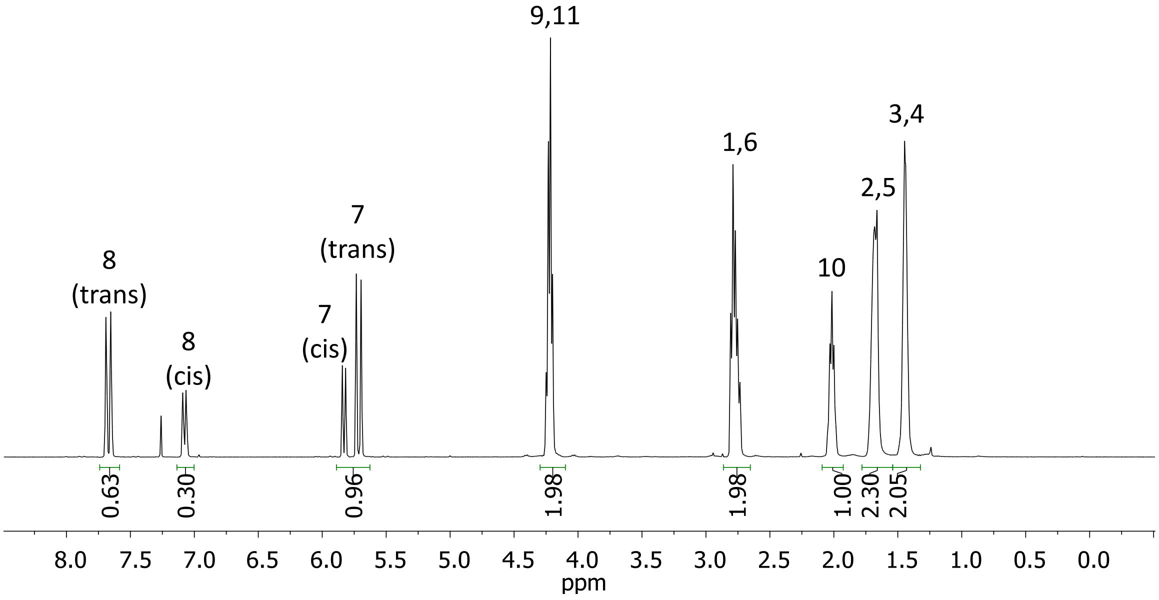

Fig. S3

^{1}H NMR spectra of C3A-C6S polymers with different %*cis* double bonds (80, 70, 53, 32%). (400 MHz, $CDCl_3$, * – Et_2O; # – DMF).

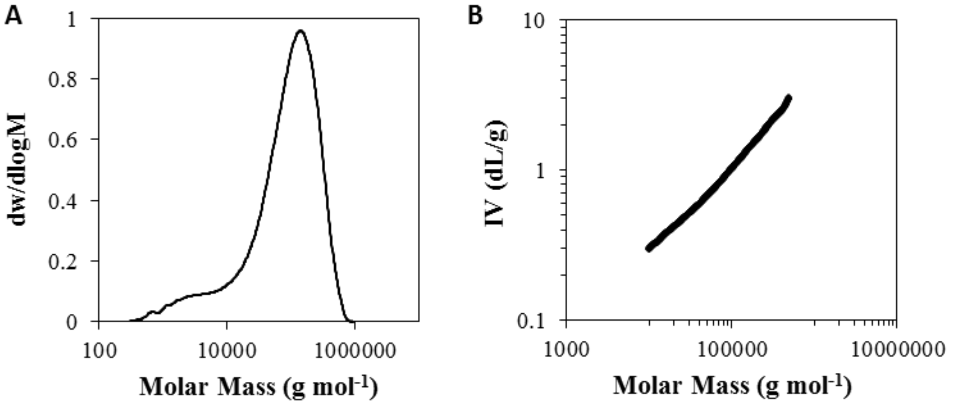

Fig. S4.

SEC analysis of 80% *cis* C_{3A}-C_{6S} (SEC $CHCl_3$ containing 0.5% v/v Et_3N). (**A**) SEC chromatogram (RI detection calibrated against polystyrene standards) M_n = 18.3 kDa, M_w = 132.4 kDa, M_p = 137.5 kDa, $Đ_M$ = 7.25; (**B**) Plot of intrinsic viscosity *vs* molar mass (K = 95.6 × 10^{-5} dL g^{-1}, α = 0.61). The α value is calculated from the gradient of the line and is consistent with a linear polymer in a theta solvent.

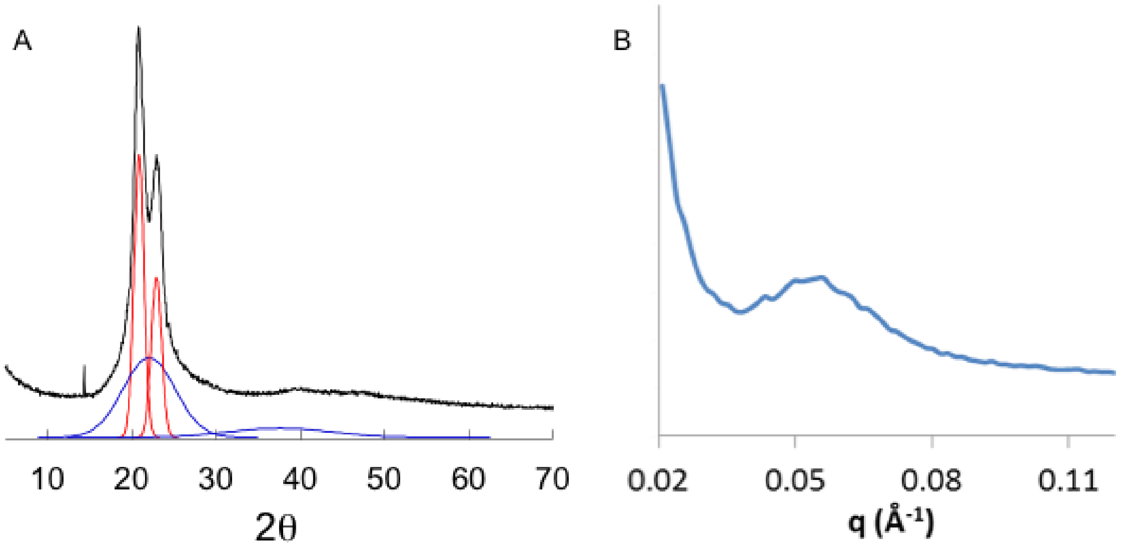

Fig. S5.

X-Ray scattering data for materials after stretching. C_{3A}-C_{6S} polymers with 80 %*cis* double bonds after stretching. (A) Wide-Angle X-Ray Diffraction shows that the material retains a high degree of crystallinity 22.2% crystallinity before stretching and 46.8% crystallinity after stretching) that is consistent with their mechanical performance; (B) Small-Angle X-Ray Scattering shows that the crystalline domain size, calculated from the q value at maximum intensity, reduces in line with that expected from the stretching of the sample.

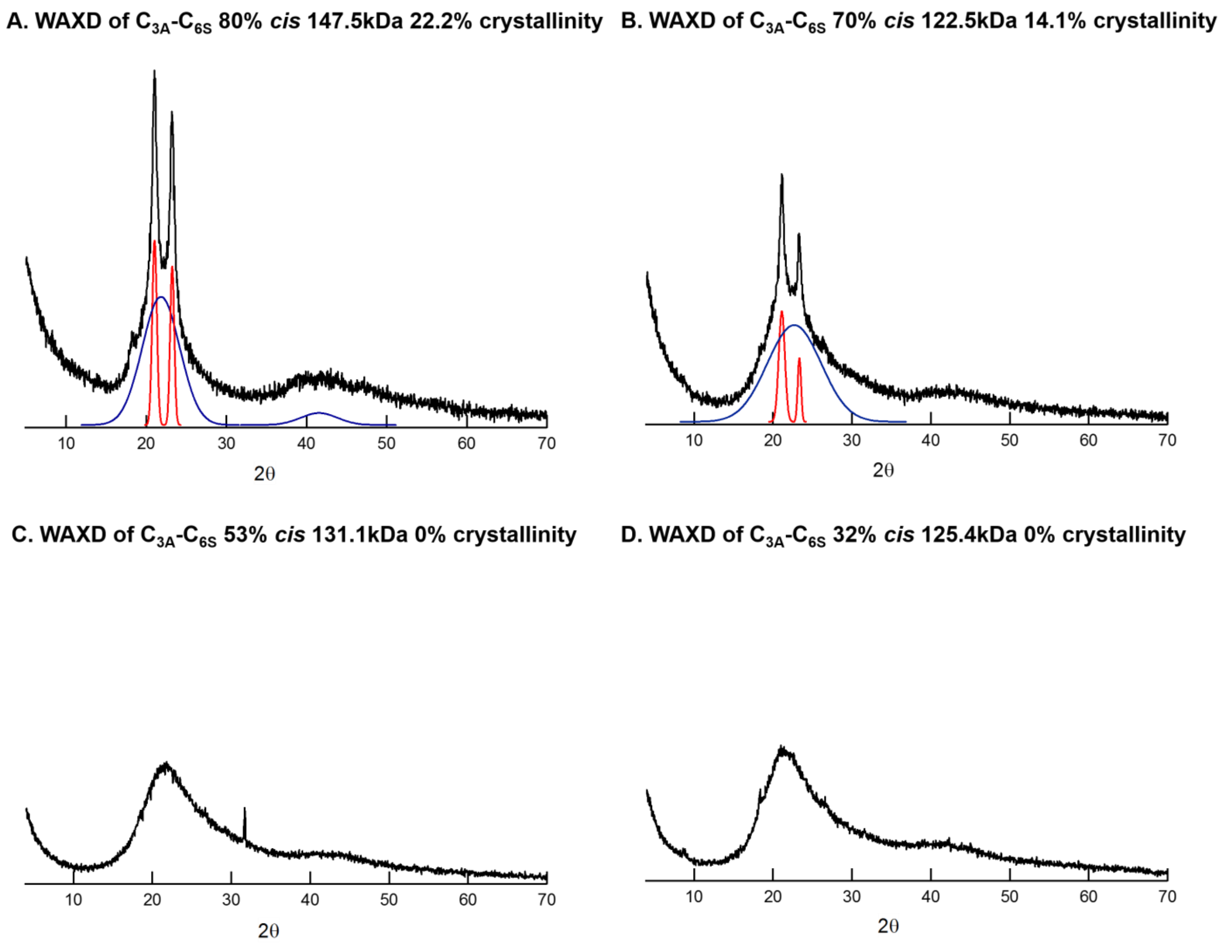

Fig. S6.

WAXD patterns of C_{3A}-C_{6S} polymers with different %*cis* double bonds (80, 70, 53, 32%) before stretching. Black lines are the original data. The red lines are the fitting of the crystalline peaks. The blue lines are the fitting of the amorphous phases. WAXD shows that C_{3A}-C_{6S} with higher %*cis* double bonds display increased crystallinity that is consistent with their mechanical performance.

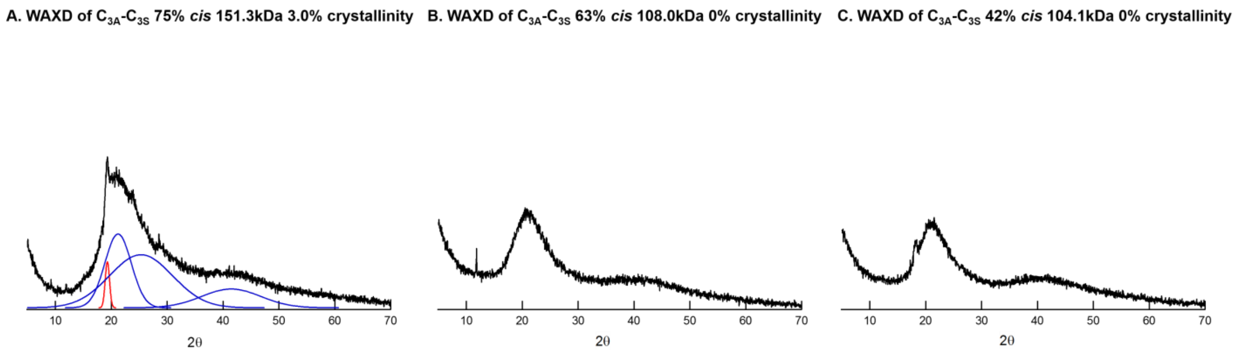

Fig. S7

WAXD patterns of C_{3A}-C_{3S} systems with different %*cis* double bonds (75, 63, 42%). Black lines are the original data. The red lines are the fitting of the crystalline peaks. The blue lines are the fitting of the amorphous phases. WAXD shows that C_{3A}-C_{3S} with higher %*cis* double bonds display increased crystallinity that is consistent with their mechanical performance.

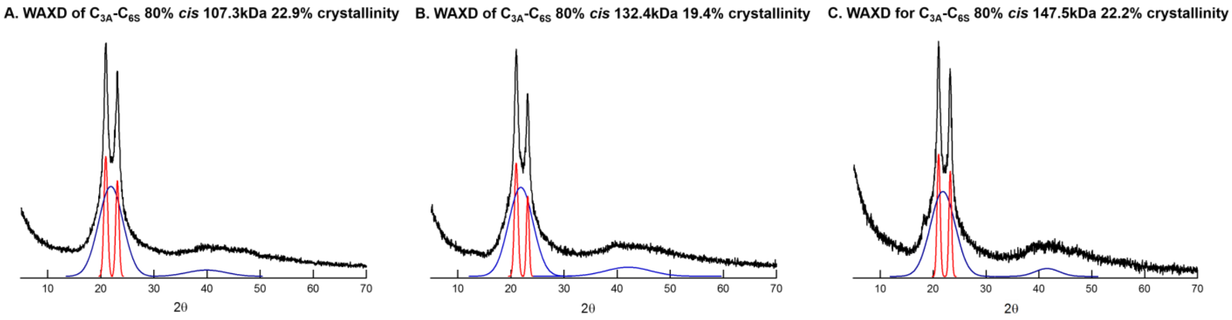

Fig. S8

WAXD patterns of 80% *cis* C_{3A}-C_{6S} systems with different molecular weights (107.3, 132.4, 147.5 kDa). Black lines are the original data. The red lines are the fitting of the crystalline peaks. The blue lines are the fitting of the amorphous phases. WAXD results show that the C_{3A}-C_{3S} polymers with the same cis% possess similar crystallinity.

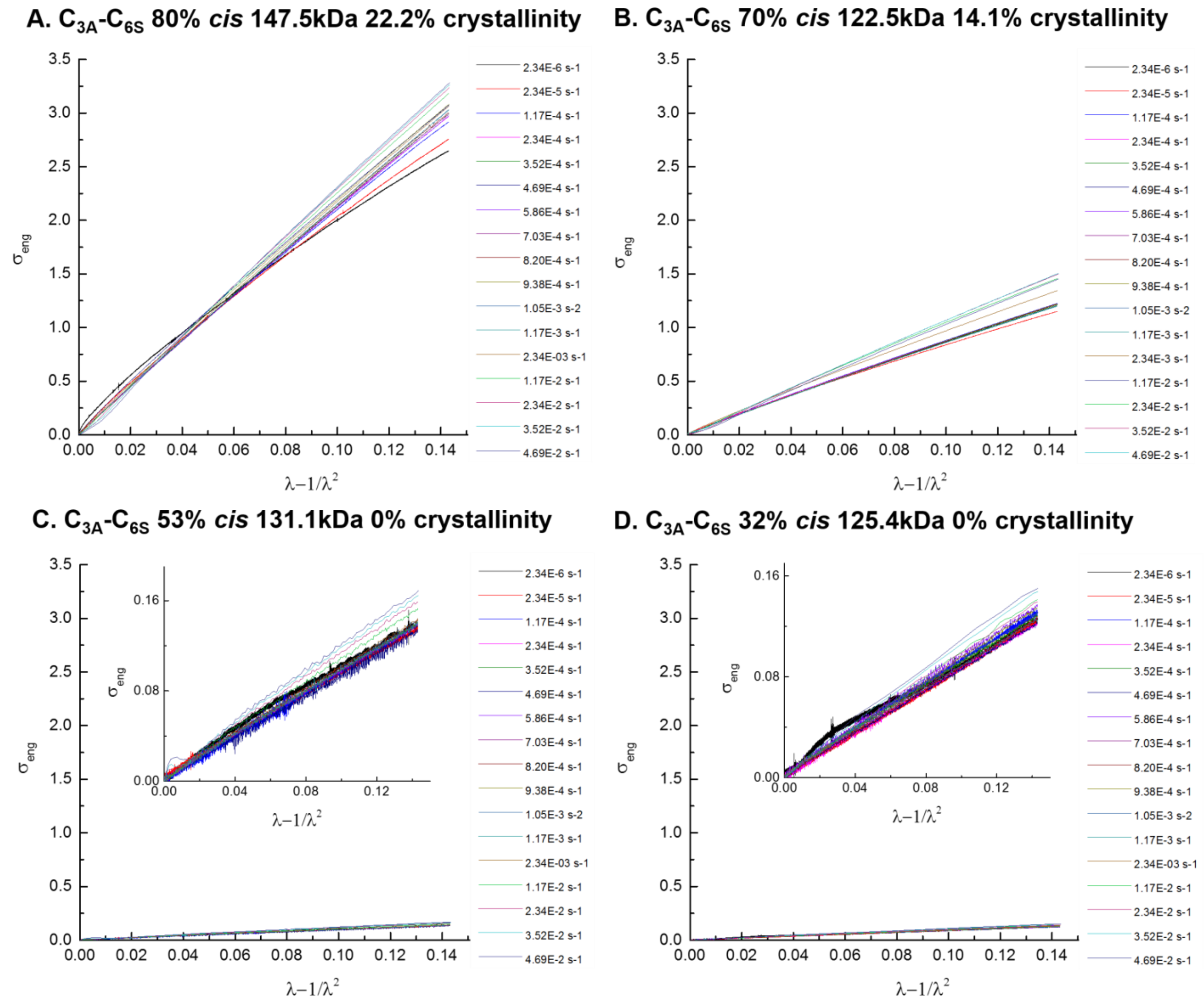

Fig. S9

Stress-Strain curves of C3A-C6S polymers with different %*cis* (80, 70, 53, 32%) at different strain rates. Expansions inset for clarity. ($\lambda = \varepsilon + 1$)

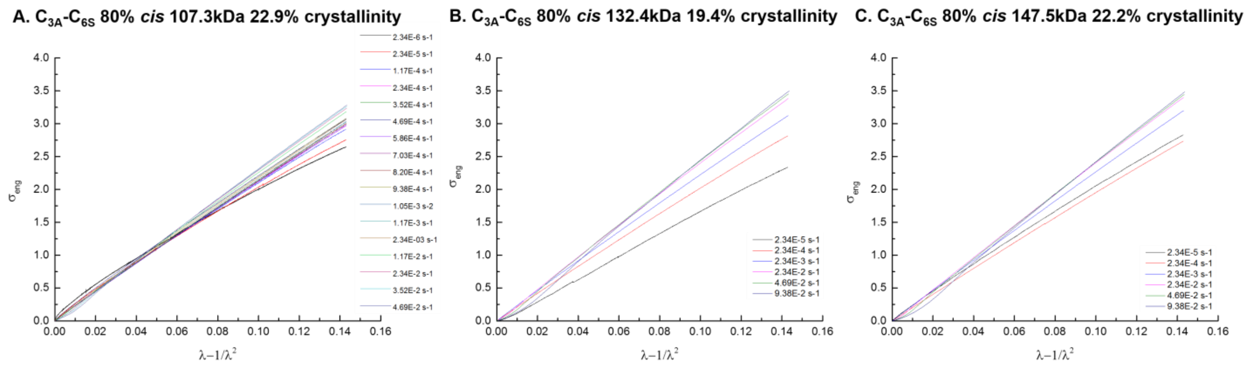

Fig. S10

Stress-Strain curves of 80% *cis* C_{3A}-C_{6S} polymers at different strain rates. ($\lambda = \varepsilon + 1$)

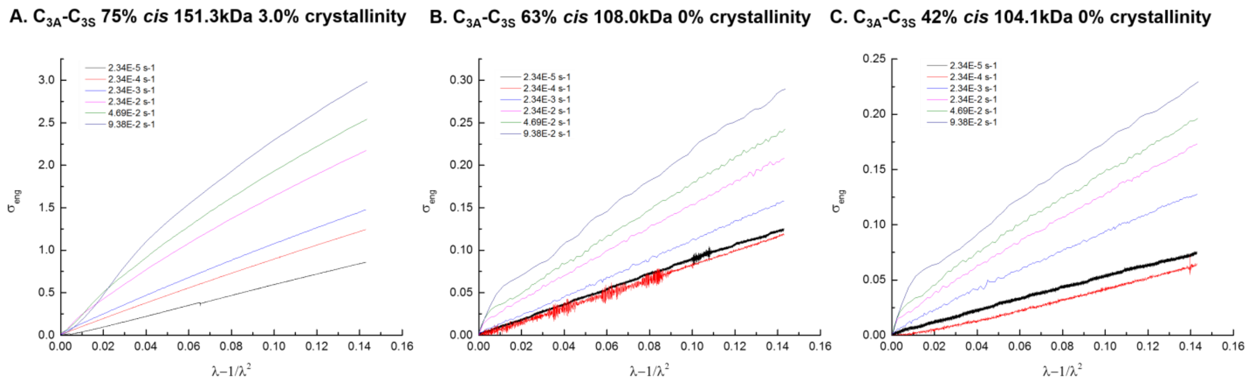

Fig. S11

Stress-Strain curves of C_{3A}-C_{3S} polymers with different %*cis* (75, 63, 42%) at different strain rates. ($\lambda = \varepsilon + 1$)

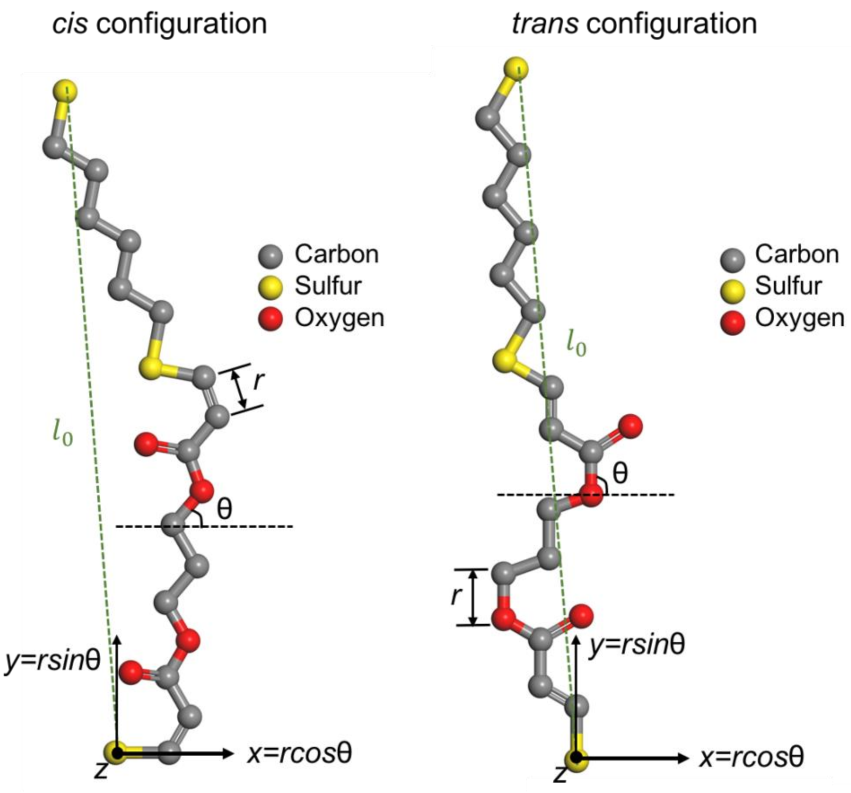

Fig. S12

Conformation of the C_{3A}-C_{6S} monomer in *cis/trans* configuration.

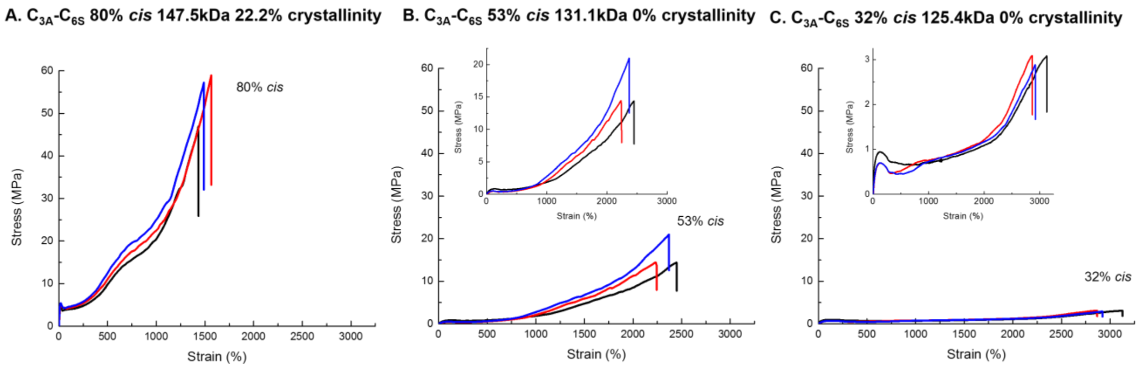

Fig. S13

Exemplar stress *vs* strain curves for C_{3A}-C_{6S} polymers with different %*cis* (80, 53, 32%) tested at 20mm/min. Data for 3 samples are shown to illustrate the reproducibility. Expansions inset for clarity. Results are listed in Table S2.

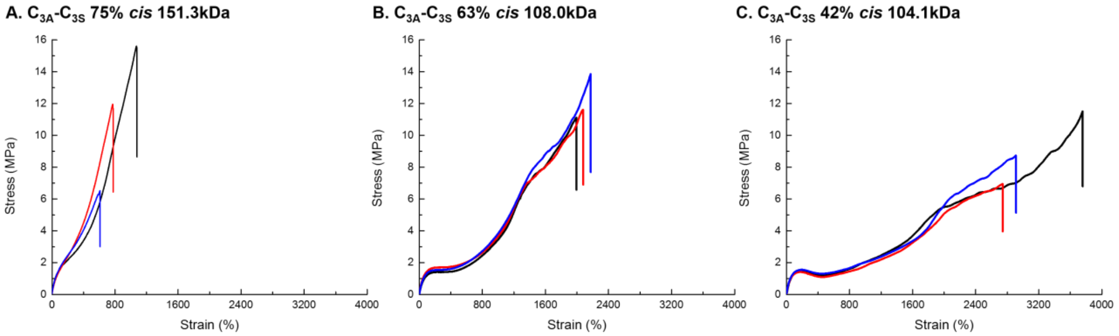

Fig. S14

Exemplar stress *vs* strain curves for C$_{3A}$-C$_{6S}$ polymers with different %*cis* (80, 53, 32%) tested at 20mm/min. Data for 3 samples are shown to illustrate the reproducibility. Expansions inset for clarity. Results are listed in Table S2.

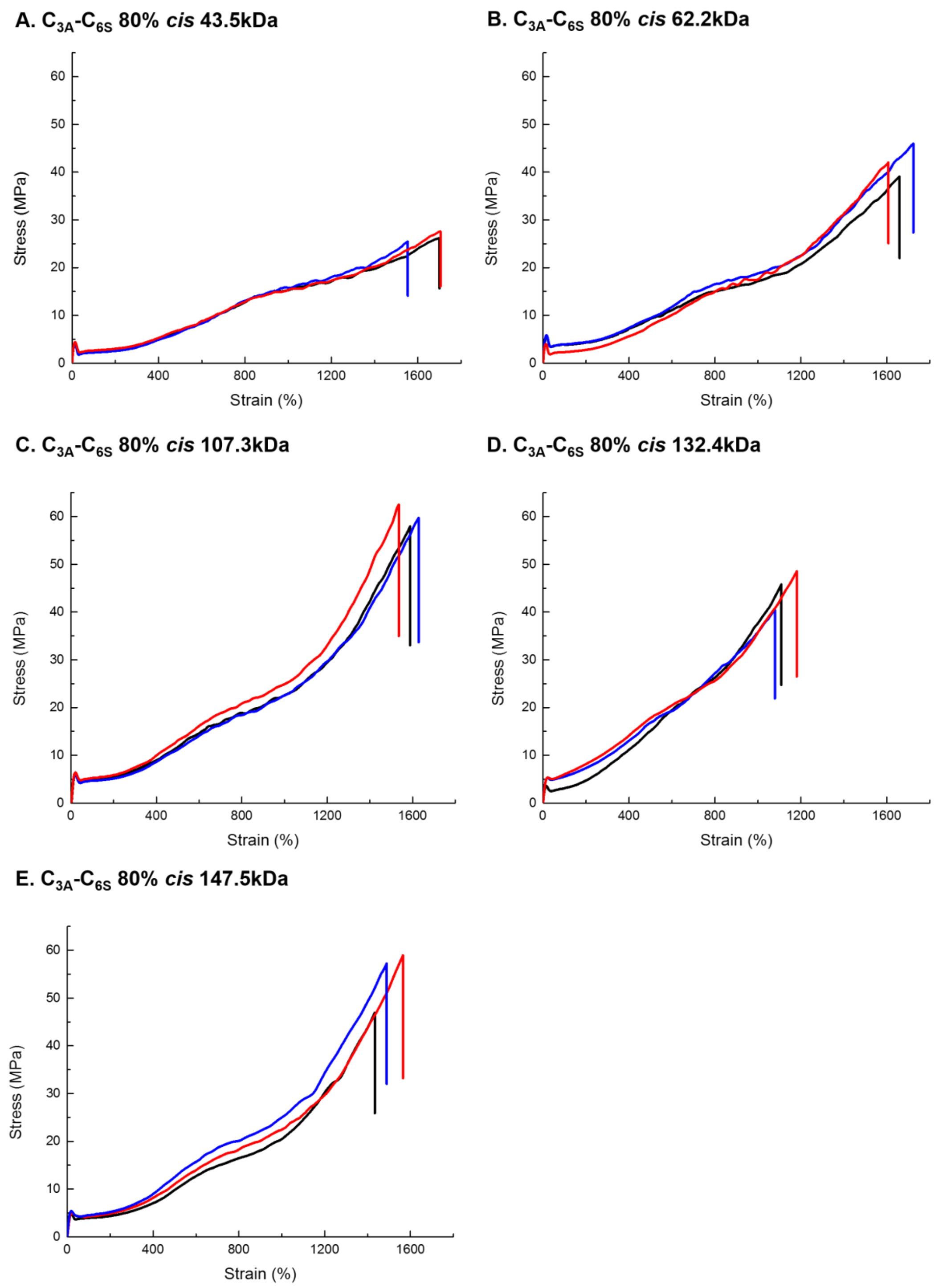

Fig. S15

Exemplar stress *vs* strain curves for C_{3A}-C_{6S} polymers with different %*cis* (80, 53, 32%) tested at 20mm/min. Data for 3 samples are shown to illustrate the reproducibility. Expansions inset for clarity. Results are listed in Table S2.

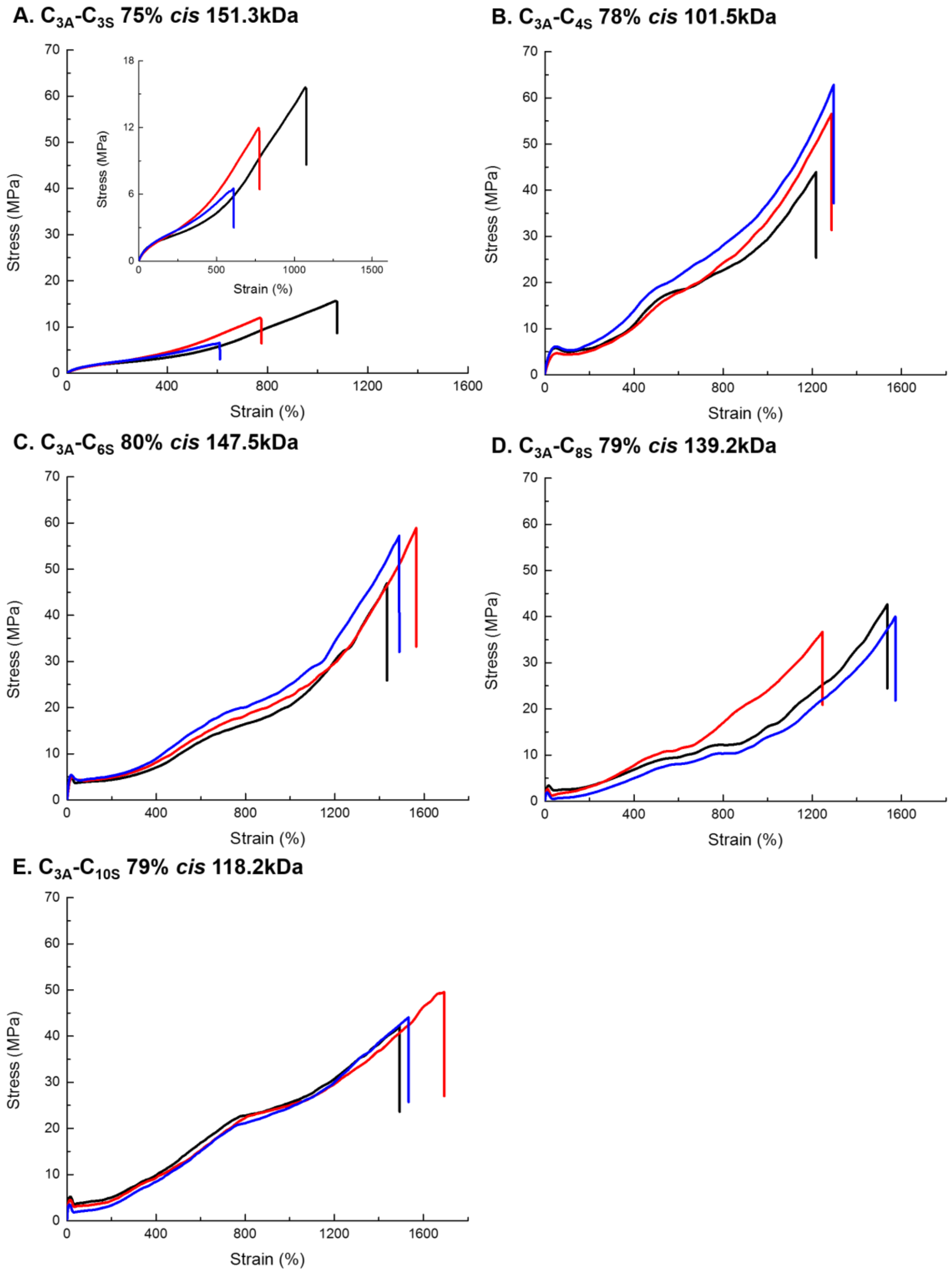

Fig. S16

Exemplar stress *vs* strain curves for C3A-CXS polymers with similar %*cis* tested at 20mm/min. Data for 3 samples are shown to illustrate the reproducibility. Expansions inset for clarity. Results are listed in Table S3.

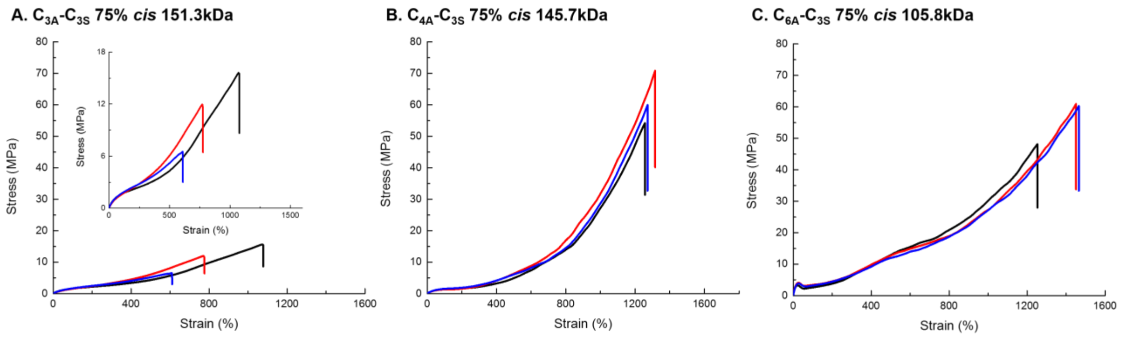

Fig. S17

Exemplar stress *vs* strain curves for C_{XA}-C_{3S} polymers with similar %*cis* tested at 20mm/min. Data for 3 samples are shown to illustrate the reproducibility. Expansions inset for clarity. Results are listed in Table S3.

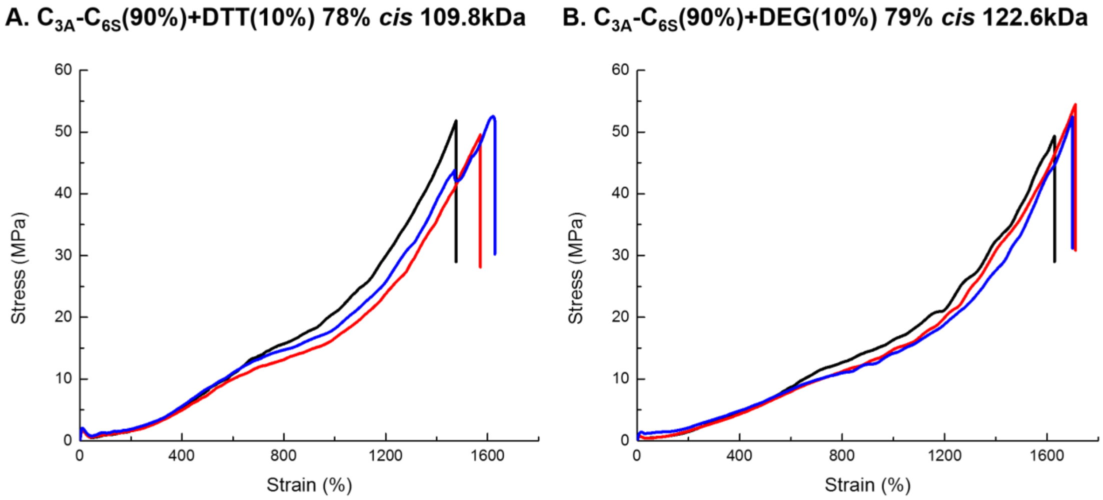

Fig. S18

Exemplar stress *vs* strain curves for C_{3A}-C_{6S}(90%)+DTT(10%) polymer and C_{3A}-C_{6S}(90%)+DEG(10%) with similar %*cis* tested at 20mm/min. Data for 3 samples are shown to illustrate the reproducibility. Results are listed in Table S3.

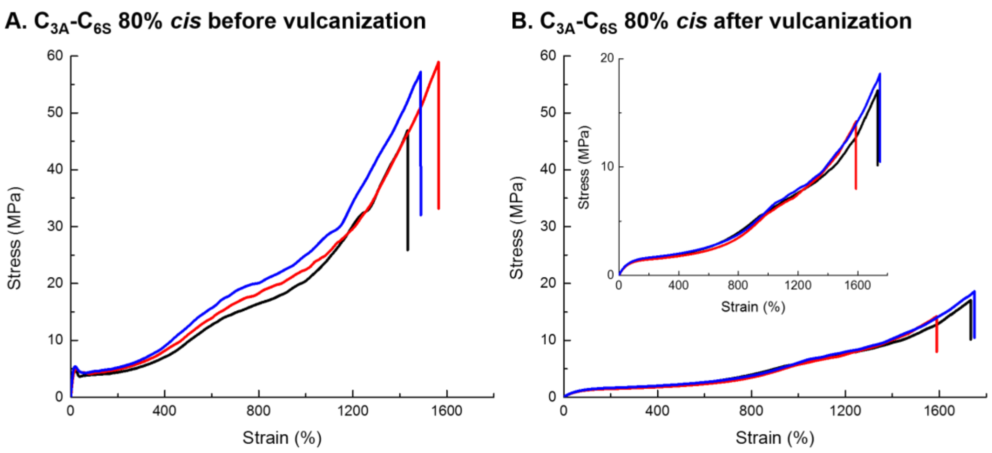

Fig. S19

Exemplar stress *vs* strain curves for 80% *cis* C_{3A}-C_{6S} polymers before and after vulcanization tested at 20mm/min. Data for 3 samples are shown to illustrate the reproducibility. Expansions inset for clarity. Results are listed in Table S3.

Table S1.

Crosshead speeds and strain rates of tensile tests.

**Crosshead Speed (mm/min)**	**Strain Rate* (s^{-1})**	**Crosshead Speed (mm/min)**	**Strain Rate* (s^{-1})**	**Crosshead Speed (mm/min)**	**Strain Rate* (s^{-1})**
0.001	2.34×10^{-6}	0.3	7.03×10^{-4}	10	2.34×10^{-2}
0.01	2.34×10^{-5}	0.35	8.20×10^{-4}	15	3.52×10^{-2}
0.05	1.17×10^{-4}	0.4	9.38×10^{-4}	20	4.69×10^{-2}
0.1	2.34×10^{-4}	0.45	1.05×10^{-3}	40	9.38×10^{-2}
0.15	3.52×10^{-4}	0.5	1.17×10^{-3}	80	1.88×10^{-1}
0.2	4.69×10^{-4}	1	2.34×10^{-3}		
0.25	5.86×10^{-4}	5	1.17×10^{-2}		

*strain rate = crosshead speed/original length

Table S2.
End-to-end distance of the C_{3A}-C_{6S} monomer in *cis/trans* configuration.

atom start	atom end	*r* *(pm)*	***cis* configuration**			***trans* configuration**		
			θ *(°)*	$r\cos\theta$ *(pm)*	$r\sin\theta$ *(pm)*	θ *(°)*	$r\cos\theta$ *(pm)*	$r\sin\theta$ *(pm)*
C	S	182	79.5	33.2	179.0	60.0	91.0	157.6
C	C	154	150.0	-133.4	77.0	130.5	-100.0	117.1
C	C	154	79.5	28.1	151.4	60.0	77.0	133.4
C	C	154	150.0	-133.4	77.0	130.5	-100.0	117.1
C	C	154	79.5	28.1	151.4	60.0	77.0	133.4
C	C	154	150.0	-133.4	77.0	130.5	-100.0	117.1
S	C	182	79.5	33.2	179.0	60.0	91.0	157.6
C	S	172	169.5	-169.1	31.3	150.0	-149.0	86.0
C	C	134	109.5	-44.7	126.3	90.0	0.0	134.0
C	C	147	49.5	95.5	111.8	150.0	-127.3	73.5
O	C	136	109.5	-45.4	128.2	90.0	0.0	136.0
C	O	143	49.5	92.9	108.7	19.5	134.8	47.7
C	C	154	120.0	-77.0	133.4	90.0	0.0	154.0
C	C	154	49.5	100.0	117.1	19.5	145.2	51.4
O	C	143	120.0	-71.5	123.8	90.0	0.0	143.0
C	O	136	60.0	68.0	117.8	150.0	-117.8	68.0
C	C	147	120.0	-73.5	127.3	90.0	0.0	147.0
C	C	134	60.0	67.0	116.0	150.0	-116.0	67.0
S	C	172	0.0	172.0	0.0	90.0	0.0	172.0
$\sum r\cos\theta$ or $\sum r\sin\theta$				-163.5	2133.6		-194.2	2212.9
$\sqrt{(\sum r\cos\theta)^2+(\sum r\sin\theta)^2}$ *(pm)*				2139.8			2221.4	

C_{3A}-C_{6S}	100% *cis*	80% *cis*	70% *cis*	53% *cis*	32% *cis*	0% *cis*
l_0 *(nm)*	2.140	2.156	2.164	2.178	2.195	2.221

Shear modulus (G) can be calculated from elastic modulus (E') by $G=E'/2(1+\upsilon)$, where υ is the Poisson's ratio. For soft elastomers, υ is 0.49. The molecular weight of strands supporting stress (M) can be calculated from network shear modulus (G) by $G \approx \rho RT/M \Rightarrow M \approx \rho RT/G$, where ρ is density (assume 1.0 g/cm^3 for all these elastomers) , R is the gas constant and T is the absolute temperature. Contour length of the chain can be determined as $L_0 = l_0 M/M_0$, where M_0 is monomer molecular weight (330.10 g/mol) and l_0 is the length of monomer in cis/trans configuration (shown in Figure 1 and results listed in Table 1). l_0 is calculated as the end-to-end distance by $\sqrt{(\sum r\cos\theta)^2+(\sum r\sin\theta)^2}$, where r is the bond length and θ is the angle between x axis and the bond.

The maximum extension ratio $\lambda_{\max}$ can be estimated (Table S3) using

$$\lambda_{\max} \approx \frac{L_0}{\sqrt{b_K L_0}} \approx \sqrt{\frac{l_0 M}{b_K M_0}} \approx \sqrt{\frac{l_0 \rho RT}{b_K G M_0}}$$

where b_K is the polymer Kuhn length. For flexible chains it varies between 0.8 and 1.4 nm.

Table S3.

Mechanical testing data and thermal analysis for C_{3A}-C_{6S} materials.

			Mechanical Properties					Thermal Properties			
M_w (kg mol^{-1})	$Đ_M$	%*cis*	E' (MPa)	ε_{yield} (%)	σ_{yield} (MPa)	ε_{break} (%)	σ_{break} (MPa)	T_g (°C)	T_c (°C)	T_m (°C)	ΔH_m (J g^{-1})
132.4	7.25	80	52.9 ± 0.6	18 ± 3	4.7 ± 1.0	1123 ± 52	44.9 ± 4.1	-1.2	45.1	81.7	-10.2
107.3	6.09	80	58.0 ± 2.0	21 ± 1	6.2 ± 0.3	1584 ± 47	60.0 ± 2.3	-1.9	50.8	81.9	-7.0
62.2	3.66	80	73.3 ± 1.4	15 ± 2	5.1 ± 1.0	1663 ± 58	42.4 ± 3.5	-1.7	40.4	82.2	-14.9
43.5	3.52	80	77.0 ± 2.6	12 ± 1	3.9 ± 0.2	1650 ± 67	27.4 ± 1.2	-1.2	40.0	80.4	-14.8
147.5	5.60	80	51.9 ± 9.6	20 ± 3	5.0 ± 0.4	1495 ± 66	54.3 ± 6.5	-1.2		75	
122.5	4.87	70	26.1 ± 2.2	28 ± 1	2.8 ± 0.3	1874 ± 94	48.3 ± 9.8	-5.1	-	-	-
131.1	3.99	53	1.5 ± 0.2	99 ± 6	0.4 ± 0.1	2252 ± 115	16.2 ± 4.2	-15.5	-	-	-
125.4	3.68	32	2.4 ± 0.2	111 ± 17	0.8 ± 0.1	2970 ± 137	3.0 ± 0.1	-16.0	-	-	-

Mechanical data were determined by tensile testing and are presented as a mean ± s.d. of three independent measurements. Thermal data is reported from DSC 2nd run. T_g, T_m and ΔH_m are obtained from heating curves and T_c from cooling curves. M_w determined by SEC analysis, %cis content determined by ^{1}H NMR analysis.

Table S4.

Mechanical testing data for materials with varied composition.

Alkyne Monomer	*Thiol Monomer(s)*	M_w (kg mol^{-1})	$Đ_M$	%*cis*	Mechanical Properties: E' (MPa)	ε_{yield} (%)	σ_{yield} (MPa)	ε_{break} (%)	σ_{break} (MPa)
C_{3A}	C_{3S}	151.3	5.14	75	2.9 ± 0.3	-	-	769 ± 262	10.7 ± 4.6
C_{4A}	C_{3S}	145.7	4.94	74	3.8 ± 0.3	-	-	1281 ± 31	61.7 ± 8.5
C_{6A}	C_{3S}	105.8	4.36	75	36.1 ± 3.1	25 ± 2	3.7 ± 0.5	1390 ± 119	56.5 ± 7.3
C_{3A}	C_{4S}	101.5	5.35	78	27.4 ± 7.8	48 ± 3	5.5 ± 0.8	1266 ± 43	54.4 ± 9.6
C_{3A}	C_{8S}	139.2	2.88	79	43.8 ± 3.3	13 ± 4	2.6 ± 0.8	1451 ± 179	39.8 ± 3.0
C_{3A}	C_{10S}	118.2	2.74	79	87.6 ± 6.3	14 ± 2	4.6 ± 0.6	1608 ± 103	46.7 ± 4.1
C_{3A}	C_{3S}	108.0	5.08	63	3.1 ± 0.2	167 ± 1	1.5 ± 0.2	2081 ± 91	12.2 ± 1.4
C_{3A}	C_{3S}	104.1	5.63	42	3.7 ± 0.4	175 ± 8	1.5 ± 0.2	3236 ± 508	9.0 ± 2.3
C_{3A}	C_{6S} (90%) + DTT (10%)	109.8	3.77	78	39.3 ± 1.9	9 ± 1	1.8 ± 0.2	1557 ± 74	51.3 ± 1.5
C_{3A}	C_{6S} (90%) + DEG (10%)	122.6	4.26	79	20.7 ± 3.5	11 ± 4	1.0 ± 0.4	1680 ± 44	52.1 ± 2.6
C_{3A}*	C_{6S}	147.5	5.60	80	2.7 ± 1.0	-	-	1690 ± 89	16.6 ± 2.2

Mechanical data were determined by tensile testing and are presented as a mean ± s.d. of three independent measurements. M_w determined by SEC analysis, %*cis* content determined by ^{1}H NMR analysis. DTT = 1,4-dithio-*d*-threitol; DEG = 2,2′-(ethylenedioxy)diethanethiol. * sample vulcanised with 1 %wt dicumyl peroxide.

Table S5.

Surface energy properties for materials with varied composition.

Alkyne Monomer	Thiol Monomer(s)	M_w (kg mol^{-1})	$Đ_M$	%cis	Before/After Stretching	γ_S^p (mJ·m^{-2})	γ_S^d (mJ·m^{-2})	γ_S (mJ·m^{-2})
C_{3A}	C_{3S}	151.3	5.14	75	Before	11.8 ± 7.8	16.1 ± 6.4	27.9 ± 14.2
					After	5.7 ± 2.0	16.6 ± 2.8	22.3 ± 4.8
C_{3A}	C_{6S}	147.5	5.60	80	Before	18.6 ± 2.2	9.7 ± 1.5	28.4 ± 3.7
					After	15.0 ± 4.2	13.2 ± 3.9	28.1 ± 8.1
C_{3A}	C_{10S}	118.2	2.74	79	Before	3.5 ± 0.7	17.3 ± 1.5	20.8 ± 2.2
					After	12.8 ± 1.6	10.5 ± 1.5	23.3 ± 3.1
C_{3A}	C_{6S} (90%) + DTT (10%)	109.8	3.77	78	Before	7.5 ± 3.2	11.2 ± 3.7	18.7 ± 6.9
C_{3A}	C_{6S} (90%) + DEG (10%)	122.6	4.26	79	Before	3.6 ± 2.1	17.6 ± 4.4	21.1 ± 6.5

γ_S^d is the dispersion component of polymer surface energy, γ_S^p is the polar component of polymer surface energy, γ_S is the overall polymer surface energy. Surface energies were determined from contact angle measurements with four different probe liquids (propylene glycol, ethylene glycol, glycerol and water) using a Goniometer and fitting the data using the Owen's equation. Data are presented as a mean ± s.d. of five independent measurements. M_w determined by SEC analysis, %*cis* content determined by ^{1}H NMR analysis. DTT = 1,4-dithio-*d*-threitol; DEG = 2,2′-(ethylenedioxy)diethanethiol.

Table S6.
Surface Tension of Liquids used for the Contact Angle Measurement

	Propylene glycol (PG)	Ethylene glycol (EG)	Glycerol (G)	Water (H_2O)
$\gamma_S^p\ (mJ/m^2)$	9.0	16.0	26.4	51.0
$\gamma_S^d (mJ/m^2)$	26.4	32.8	37.0	21.8
$\gamma_S (mJ/m^2)$	35.4	48.8	63.4	72.8

References

1. B. M. Mandal, *Fundamentals of Polymerization*. Singapore (World Scientific Publishing Co., 2013).
2. V. X. Truong, A. P. Dove, Organocatalytic, Regioselective Nucleophilic "Click" Addition of Thiols to Propiolic Acid Esters for Polymer-Polymer Coupling. *Angew. Chem. Int. Ed.* **52**, 4132-4136 (2013).
3. I. M. Ward, J. Sweeney, *Mechanical Properties of Solid Polymers*. (Wiley-Interscience, London, 1971).
4. A. Carre, Polar interactions at liquid/polymer interfaces. *J. Adhes. Sci. Technol.* **21**, 961 - 981 (2007).
5. A. F. Toussaint, P. Luner, The wetting properties of grafted celulose films. *J. Adhes. Sci. Technol.* **7**, 635 - 648 (1993).
6. C. J. van Oss, *Interfacial Forces in Aqueous Media, 2nd Ed.*, (CRC Press, Boca Ranton, FL, 2006).
